# Tuning the Properties of Multi‐Stable Structures Post‐Fabrication Via the Two‐Way Shape Memory Polymer Effect

**DOI:** 10.1002/advs.202308903

**Published:** 2024-03-17

**Authors:** Giada Risso, Max Kudisch, Paolo Ermanni, Chiara Daraio

**Affiliations:** ^1^ Laboratory of Composite Materials and Adaptive Structures, Department of Mechanical and Process Engineering ETH Zürich Leonhardstrasse 21 CH‐8092 Zürich Switzerland; ^2^ Engineering and Applied Science California Institute of Technology 1200 E. California Blvd. Pasadena CA 91125 USA

**Keywords:** adaptive structures, multi‐stability, reprogrammable structures, thin‐ply composite

## Abstract

Multi‐stable elements are commonly employed to design reconfigurable and adaptive structures, because they enable large and reversible shape changes in response to changing loads, while simultaneously allowing self‐locking capabilities. However, existing multi‐stable structures have properties that depend on their initial design and cannot be tailored post‐fabrication. Here, a novel design approach is presented that combines multi‐stable structures with two‐way shape memory polymers. By leveraging both the one‐way and two‐way shape memory effect under bi‐axial strain conditions, the structures can re‐program their 3D shape, bear loads, and self‐actuate. Results demonstrate that the structures' shape and stiffness can be tuned post‐fabrication at the user's need and the multi‐stability can be suppressed or activated on command. The control of multi‐stability prevents undesired snapping of the structures and enables higher load‐bearing capability, compared to conventional multi‐stable systems. The proposed approach offers the possibility to augment the functionality of existing multi‐stable concepts, showing potential for the realization of highly adaptable mechanical structures that can reversibly switch between being mono and multi‐stable and that can undergo shape changes in response to a change in temperature.

## Graphical abstract

Cutting‐edge assemblies of multi‐stable structures with two‐way shape memory polymers allow on‐demand transformations of shape and stiffness of the structures. The structures' shape can be re‐programmed to bear loads or suppress multi‐stability. Results show potential for the realization of adaptable mechanical systems that reversibly switch between mono and multi‐stable and self‐actuate in response to temperature changes.

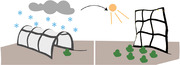

## 1 Introduction

Mechanical instabilities, once avoided by engineers and researchers, have now become a powerful design principle.^[^ ^1^, ^2^, ^3^, ^4^ ^]^ By leveraging elastic buckling, engineers created adaptive systems that exhibit complex behaviors, such as self‐assembly, shape‐changing, and reversible deformation.^[^ ^5^, ^6^, ^7^, ^8^ ^]^ Instabilities in structures can be induced by incorporating into anisotropic geometries soft and active materials such as shape memory polymers (SMP),^[^ ^9^, ^10^ ^]^ liquid crystal elastomers,^[^ ^11^ ^]^ hydrophilic materials,^[^ ^12^ ^]^ and hydrogels.^[^ ^13^ ^]^ Such materials undergo pre‐defined shape changes in response to external stimuli, leveraging local deformations. A common strategy to induce local buckling is to mechanically create internal stresses.^[^ ^14^, ^15^ ^]^ Once released, such stresses can generate complex 3D shapes in a controlled manner.^[^ ^16^ ^]^ These design principles have been employed for a wide spread of applications such as actuators,^[^ ^17^, ^18^ ^]^ soft mechanical diodes,^[^ ^19^ ^]^ and biomedical tissues,^[^ ^20^ ^]^ offering simple manufacturing routes and large shape alteration.

Most solutions, however, can generate only one target 3D shape or are based on soft materials that are not well suited for structural purposes. In addition, structures should maintain a desired shape without the need for a continuous power supply, and adapt their stiffness to perform various tasks. To include all such properties, multi‐stability has been proposed as a possible solution.^[^ ^21^, ^22^, ^23^, ^24^, ^25^, ^26^ ^]^ Multi‐stable structures are characterized by the presence of multiple minima in their energy landscape. They can intrinsically hold their stable states, requiring a preset energy to transition between stable configurations. Such structures have been shown to allow for multiple, rapid, and reversible shape alterations.^[^ ^27^, ^28^ ^]^ Despite such advantages, the range of allowed 3D shapes is dictated by the structure's sub‐constituents and cannot be altered post‐fabrication. Commonly, the stable states and stiffness response to external loads in each stable configuration of such structures are fixed, decreasing their adaptive properties and implementation in real‐world applications.

Recent work has focused on the realization of structures whose mechanical properties can be tailored post‐fabrication, also called programmable structures.^[^ ^29^ ^]^ For example, bi‐stability has been used to demonstrate the tunability of the response of meta‐structures under compression, or to induce chirality variations.^[^ ^29^, ^30^ ^]^ Other authors proposed using jamming^[^ ^31^, ^32^, ^33^, ^34^ ^]^ or lateral confinement^[^ ^35^ ^]^ to enable variations in stiffness. In addition, turning on and off multi‐stability has been realized to avoid undesired snapping of the structures while maintaining the shape transformation property.^[^ ^36^ ^]^ To date, no solution has demonstrated the possibility of realizing fully programmable multi‐stable structures, where locations of the minima in the energy landscape can be tailored according to the programming. Specifically, programmable multi‐stability would allow structures to assume new stable shapes and to exhibit a variable stiffness response to external loading.

**Figure 1:**
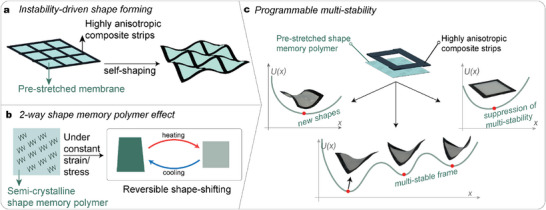
Design approach. a) Elastic instability of thin membranes generates 3D shaping of thin composite strips bonded with pre‐stretched membranes. b) Shape memory polymers induce stresses when they are recovering their original shape. c) Combining thin composite strips with pre‐stretched shape memory polymer membranes enables the realization of new meta‐structures, whose multi‐stability can be turned on and off reversibly, and whose deformations can be programmed post‐fabrication via constrained crystallization.

To program multi‐stable systems, in this work we combine two design approaches: i) The introduction of prestresses in soft members, to induce buckling and ii) The use of polymers exhibiting both one‐way and two‐way shape memory effects (1w‐SME and 2w‐SME), to control stress distribution (**Figure** **1**). Earlier work demonstrated that pre‐stressed soft membranes can induce buckling in thin composite strips and create 3D structures from a flat assembly.^[^ ^37^ ^]^ With this method is possible to manufacture multi‐stable structures only if the composite strips are made by employing highly anisotropic materials with a high ratio of bending to torsional stiffness, realizing periodic meta‐structures with many stable states.^[^ ^38^ ^]^ The interaction between the soft, pre‐stressed membrane and the stiff, composite strips underpins the boundary‐free 3D shape‐forming process. In such systems, modulation of internal stresses could enable control over shape formation.

The 2w‐SME in polymers results in a reversible cycling between two physical states, in response to an external stimulus.^[^ ^39^, ^40^ ^]^ Specifically, semi‐crystalline SMPs under constant strain conditions are characterized by states with distinct mechanical properties, producing changes in internal stresses in response to changes in temperature. For example, earlier studies compared a uniaxially pre‐stressed, semi‐crystalline polymer strip sample with a second sample that had been chemically cross‐linked to form an SMP. Both samples were fixed at constant strain and the stress was monitored during a thermomechanical heating/cooling cycle.^[^ ^41^ ^]^ During the heating cycle, the SMP sample exhibited a rise in stress upon melting due to shape memory induced contraction. During cooling from the melt state, a drop in stress was observed upon crystallization due to the 2w‐SME. Neither effect was observed in the sample that had not been cross‐linked. Together, these effects formed a closed temperature response loop in the SMP sample that was utilized to construct an actuator that exhibited reversible curvature changes over multiple heating/cooling cycles.

In our work, we extend for the first time the use of two‐way shape memory polymers to a 2D system, in which the drop in internal stress upon initial stages of melting or crystallization translates to the programmability of the structure's shape (Figure 1c). Our approach leads to an instability‐driven shape morphing of composite flat networks by pre‐stretching semi‐crystalline SMP membranes to realize a new class of meta‐structures whose shape, multi‐stability behavior, and stiffness can be tuned post‐fabrication. We combine the stimulus‐driven shape‐change principles of SMPs with the design of meta‐structures with many stable states, to switch on‐ and off‐ the stability of structures, control their overall stiffness, and induce shape changes into different, stable configurations.

## 2 Results

### 2.1 Design and Multi‐Stability of a Single Cell

Previous investigations realized multi‐stable frames by combining a bi‐axially pre‐stretched, thermoplastic polyurethane (TPU) membrane with flat, fiber‐reinforced, polymer composite (FRP) strips.^[^ ^38^ ^]^ It was demonstrated that to have multi‐stability it is necessary to employ strips with a high ratio of torsion to bending stiffness. Therefore, to ensure the shape forming and guarantee the multi‐stability of the frame, in this work we employ highly anisotropic fiber‐reinforced composite laminates for the strips (details in Section 4) and we fabricate in‐house a tough, shape memory membrane.

We selected poly(*cis*‐cyclooctene) (PCOE) as the shape memory polymer to fabricate the membrane based on a number of considerations, including ease and scalability of the synthesis, comparable mechanical properties to those of the TPU foil,^[^ ^37^ ^]^ a practical and tunable actuation temperature, and demonstrated evidence of 2w‐SME behavior.^[^ ^42^ ^]^ Finally, a suitable membrane material must be able to exhibit sufficient actuation force to engage the buckling in the strips necessary to induce the shape‐forming. The synthesis employed in previous studies,^[^ ^42^ ^]^ in which the PCOE is crosslinked using the thermal free radical decomposition of organic peroxides, was adapted to our purpose and scaled to a batch size of 50 g, with dibenzoyl peroxide (2.5 wt. % relative to PCOE) selected as a cross‐linker.

The membrane's chemical composition was selected to achieve a Young's modulus of 62 MPa at room temperature, which is in the same order of magnitude as the TPU foil (43 MPa^[^ ^37^ ^]^). The uniaxial tensile testing data is available in the Section S8 (Supporting Information). The membrane has a thickness between 170 and 180µm and it is designed to undergo purely elastic deformations at strain levels up to 30%–40%. The SMP membrane has a melting temperature, *T*_*m*_ = 49.2 °C, and a crystallization temperature, *T*_*c*_ = 28.8 °C, as measured by Differential Scanning Calorimetry (DSC; See Section S5; Supporting Information). Both *T*_*m*_ and *T*_*c*_ are above room temperature, *T*_*r*_, as is necessary to realize stimuli‐responsive behavior with heating without the requirement of additional cooling or excessive temperatures. The glass transition temperature, *T*_*g*_ = ~ − 70 °C, as measured by dynamic mechanical analysis (See Figure S13, Supporting Information) is too low to be relevant for triggering shape memory in the present study. In addition, the membrane was found to exhibit outstanding actuation performance in a weightlifting test (See Section S10, Supporting Information). In fact, it shows a specific work of 0.71 *Jg* ^−^^1^, measured by a pre‐stretched strip of the membrane lifting a weight of mass 104,200 times its own. Notably, this specific work is similar in magnitude to the specific work (i.e., up to 2 *Jg* ^−^^1^) measured in testing a material that features among the highest energy density of reported actuators developed in a previous work.^[^ ^43^ ^]^ Further details about the synthesis and cross‐linking process are given in the Supporting Information (Sections S1, S2, S9, and S11, Supporting Information).

**Figure 2:**
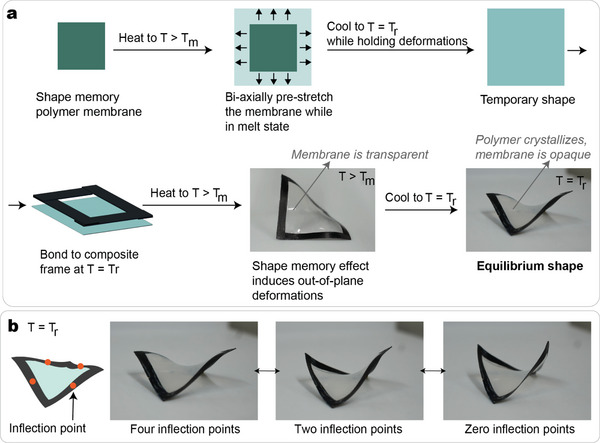
Multi‐stable single cells: manufacturing and boundary‐free behavior. a) Schematic of the main steps to manufacture a multi‐stable hybrid frame. b) The composite frame is multi‐stable at room temperature. The three main stable states of the structure are illustrated. Each strip of the structure has a width *w* = 5mm and length *L* = 50mm. The pre‐stretching of the membrane is ε=25%. T = 60 °C > T_*m*_ (49.2 °C) and *T*_*r*_ = 21 °C.

After the polymer is manufactured, it is molded into its equilibrium shape (i.e., a thin membrane) and cross‐linked. The composite frames are assembled as illustrated in **Figure** **2a**. First, the SMP is melted by applying a temperature above *T*_*m*_ of 60 °C and then equi‐biaxially pre‐stretched. The membrane is held in its pre‐stretched state while being cooled down to *T*_*r*_ which is below *T*_*c*_. Afterward, the constraints are removed and the composite frame is bonded to the SMP. By applying a temperature above *T*_*m*_ of 60 °C, the crystalline domains in the SMP membrane melt, triggering a shape recovery that induces internal stresses. The internal stresses, in turn, lead to the buckling of the composite strips. The temperature of 60 °C was selected here as it both exceeds the melt transition as measured by DSC (See Figure S5, Supporting Information) and represents the onset of the rubbery plateau indicating complete melting as measured by dynamic mechanical analysis (DMA, See Figure S12, Supporting Information). Upon cooling to room temperature, the equilibrium shape of the structure is reached. We will refer to this step as boundary free shape‐forming as during the shape forming no external forces or displacements are imposed on the frame during the temperature change. The manufactured frame has three stable states, as depicted in Figure 2b. Following the notation utilized in previous studies,^[^ ^38^ ^]^ the three stable states are labeled as states with four, two, and zero inflection points, respectively. Video S2 (Supporting Information) shows the boundary‐free shape forming of a frame and its multi‐stability at room temperature.

**Figure 3:**
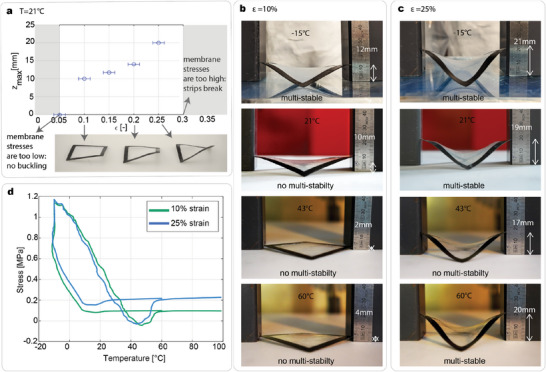
Effect of pre‐stretching and temperature on the multi‐stability. a) The out‐of‐plane deformation of the strips increases with the pre‐stretching of the membrane. b) Effect of the temperature on the out‐of‐plane deformation of the frame manufactured with ε=10%. The out‐of‐plane deformations are reported for different temperatures. c) Effect of the temperature for a frame manufactured with ε=25%. d) Stress versus temperature at the indicated constant strain values for the PCOE membrane sample. Thermal cycling begins at 60 °C, followed by cooling to −15 °C and heating to 100 °C.

The membrane pre‐stretching strongly affects the out‐of‐plane deformations of the frames, as well as its multi‐stability behavior. An experimental investigation is performed by manufacturing frames with a pre‐stretching ε = [5, 10, 15, 20, 25, 30]%. The layup and geometry of the frame and the thickness of the membrane are kept identical to those of the structure in Figure 2. As observed in **Figure** **3**a, the magnitude of the pre‐stretching of the membrane significantly affects the deformations of the frame. For the limit case of ε=5%, the energy pre‐stored in the membrane is not sufficient to induce the buckling of the strips, while for ε=30% the shape memory forces transmitted from the membrane to the frame were too high, and, during the shape‐forming, two consecutive strips delaminated at their intersection. Figure 3a reports the *z*_*max*_, defined as the maximum out‐of‐plane deformation from the plate of two opposite corners of the frame. Photographs of the frames with ε=10%, 20%, and 25% are also reported. The frames with ε=10% and 15% are not multi‐stable and show very low out‐of‐plane deformations, the strips do not show an inflection point, and they rather resemble a stable state with zero inflection points. The frame with ε=20% presents four inflection points, though no multi‐stability is observed. Only the frame with 25% pre‐stretching is observed to be multi‐stable at room temperature. The out‐of‐plane deformation *z*_*max*_ increases with the pre‐stretching^[^ ^37^ ^]^ and enhances the multi‐stability of the structures.^[^ ^38^ ^]^ The uncertainty of the experimental data stems from the imprecision of the pre‐stretching of the membrane, which is performed manually.

### 2.2 Temperature‐Dependent Multi‐Stability and Deformations

When a frame in its equilibrium shape is heated to 60 °C, the membrane melts, and a change in the out‐of‐plane deformation of the frame is observed. Afterward, if cooled to room temperature, the frame returns to its equilibrium shape dictated by a partial shape memory recovery of the SMP membrane (see Figure 3b,c second and last rows). This process can be repeated in theory an infinite number of times and is driven by the 2w‐SME. We performed a repeatability test over ten cycles observing no variation in the out‐of‐plane deformations (see details in the Section S3, Supporting Information). It is worth emphasizing that this reversible change in shape as a function of temperature comprises a cycle that could be engineered to achieve a desired actuation outcome by tailoring the unit cell design or its mode of incorporation into a larger structure, although doing so is beyond the scope of the current study. In previous studies, it has been demonstrated that the 2w‐SME occurs when the polymer is subject to constant stress or constant strain conditions. The alignment of crystallites along the stress axis is observed, resulting in a stress drop during crystallization that enables reversible thermomechanical cycles.^[^ ^42^, ^44^ ^]^ Here, we show that this 2w‐SMP effect is also enabled by a bi‐axial strain condition. The internal stresses of the membrane vary as a function of temperature, resulting in temperature‐dependent structure behavior. For the frame with ε=25%, the shape memory contraction upon melting directly relates to a slight increase of the out‐of‐plane deformations. For the structure with ε=10%, the *z*_*max*_ decreases if compared to room temperature. This is explained because the membrane in the melt state is much softer (Young's Modulus *E* = 0.92 MPa) compared to the crystallized state (Young's Modulus *E* = 62 MPa). Therefore, the contraction upon melting of the membrane with a pre‐strain of 10% is not sufficient to induce a significant buckling of the strips due to the low stiffness of the membrane itself. Only for high pre‐stretching values, the contraction upon melting of the membrane is sufficient to realize a multi‐stable frame in the melt state. Results of the tensile tests at 10, 22, 42, and 60 °C are reported in the Section S8 (Supporting Information).

Together with the deformations, the multi‐stability of the hybrid frames changes with temperature. Figure 3 illustrates the out‐of‐plane deformations of the frames with ε=10% and 25% at the temperatures of *T* = [‐15, +21, +43, +60] °C. Both structures show an increase in deformations and are multi‐stable at T = −15 °C, while they possess very low deformations at T = +43 °C. Particularly, the 10% frame has four inflection points at ‐15 °C and is almost fully flat at 43 °C. By increasing the temperature from 43 °C to 60 °C, the deformations increase for both frames.

To understand how for different pre‐strains and temperatures the multi‐stability and deformations of the structure change, a thermomechanical test is performed on the SMP membrane with a Dynamic Mechanical Analyzer (DMA; See Section S6, Supporting Information for further details). In this test, a sample consisting of a rectangular strip of the cross‐linked PCOE membrane is first heated to 60 °C, then a defined uniaxial strain is imposed and maintained constant for the rest of the experiment. Next, the sample is cooled from 60 to ‐10 °C, while the stress is monitored continuously as depicted in Figure 3d. Stress/temperature curves are highlighted for strain values of 10% and 25%, while additional curves for other strain values are reported in the Section S6 (Supporting Information). It can be observed that a stress drop occurs upon crystallization as the temperature drops below *T*_*c*_, consistent with the observations of previous studies^[^ ^41^, ^44^ ^]^ and that a change in the slope of the curve is observed around 8 °C, after which the stresses rapidly increase. The previous works explained these changes as a two‐stage crystallization process. In the first stage, a population of crystallites forms that are oriented along the axis of the applied stress, and their reorientation results in the stress drop. This stage can be thought of as the origin of the 2w‐SME, as these changes in the SMP internal structure prepare it for the next heat induced shape memory cycle, analagous to the crystallization induced elongation observed under constant stress conditions.^[^ ^42^ ^]^ Next, the stress increases due to the increase of Young's modulus of the crystallized polymer that accompanies complete crystallization as a population of randomly oriented crystallites form, defining the temporary shape of the SMP. Thereafter, the sample is heated from ‐10 to 100 °C. The stresses drastically decrease upon the initial stage of melting. At around 43 °C the minimum in the stress curves is observed, consistent with the onset of melting. As the temperature continues to increase, the strain increases as the samples recover their initial stress state once fully melted, completing a 2w‐SME stress loop. The magnitude of the shape memory contraction force exerted upon complete melting increases with the degree of pre‐strain, consistent with the increased stress magnitude observed as a plateau at temperatures *T* >T_*m*_ in Figure 3d (See Figure S14, Supporting Information for additional levels of pre‐strain). This difference in contraction force underpins the greater degree of change observed in the out‐of‐plane deformation as a function of temperature in the 10% pre‐strain case as the internal stresses are near the threshold required to achieve buckling in the FRP frame.

The two curves of Figure 3d show a shift if compared to one another. The temperature corresponding to the minimum stress upon melting is higher with lower strains, and the stress drops upon crystallization are more pronounced for higher strains. For structures fabricated with membranes at 10% and 25% pre‐strain is it observed that at around 43 °C the minimum in the out‐of‐plane deformations is occurring, concurrent with a loss of multi‐stability. This corresponds well to the stress minimum of Figure 3d; the membrane does not exert sufficient forces on the FRP strips to enable multi‐stability at 43 °C. This loss of multi‐stability is part of the 2w‐SME loop, and the corresponding change in shape occurs reversibly with each heating phase. Figure 3d also shows a pronounced increase of stress for very low temperatures. This results in higher deformations for the frames as illustrated in Figure 3 (first row). For the ε=10% case, the increase of the stresses results in turning on the multi‐stability. This drastic increase in stresses derives from the increase in stiffness of the material that occurs when cooling the sample from room temperature to −15°C. Tensile tests of membrane samples at different temperatures were performed, as well as DMA, to monitor the changes in stiffness as a function of temperature. In DMA, the tensile storage modulus of the cross‐linked PCOE membrane sample was found to increase significantly from 60–70 MPa at 20–25°C to 170.8 MPa at −15°C). Additional results are given in the Sections S6 and S8 (Supporting Information) for DMA and tensile testing, respectively, including full experimental data with strains [10,15,20,25,30]%.

### 2.3 Temperature Change as a Means for Actuation

**Figure 4:**
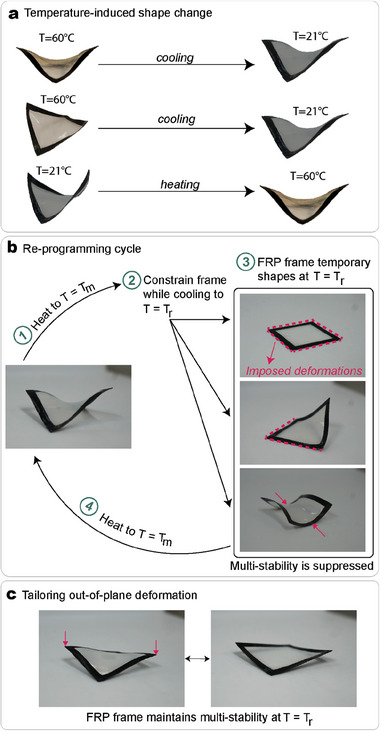
Self‐actuation and programming of composite frames. a) Changing the temperature of a multi‐stable structure induces a change of shape. Examples are reported in the Video S3 (Supporting Information). b) Steps utilized to impose a new stable state. By imposing displacements to the frame during crystallization, is possible to flatten one or multiple strips of the frame and to realize a saddle shape. c) The out‐of‐plane deformation of the structure can be increased/decreased by imposing external constraints during crystallization.

During the boundary‐free shape forming, the frame with 25% pre‐stretching shapes into the stable state with four inflection points, and once cooled at room temperature it maintains the same stable shape. The stable state with four inflection points is the one that is observed at any temperature between −15 and 60 °C. By changing the temperature of the frame in a state with two or zero inflection points, the frame snaps to its minimum energy level, thus to the state with four inflection points. Two examples of temperature‐induced change of shape are illustrated in **Figure** **4**a. One example shows that the frame in the state with zero inflection points at 60 °C when cooled down to room temperature, will snap to the state with four inflection points. The second example shows that an identical change of shape (from zero to four inflection points) can be induced by heating a frame from 21 °C to melt.

These shape transitions are validated with experiments. Figure S3 (Supporting Information) illustrates snapshots from an experiment where the shape transition triggered by the application of heat is occurring, as in the case schematized in Figure 4a. The corresponding video can be found in the Supporting Information as Video S3 (Supporting Information). The snapping of the two corners occurs one after the other one. Moreover, it is implied that the same shape change can be triggered when snapping a frame from a stable state with two inflection points to the one with four. These observations demonstrate that the actuation between stable states can be performed in response to a change in temperature. We note that the reverse actuation, where the state with four inflection points changes to another stable state, does not occur. Therefore, the actuation in this section results from the 1w‐SME.

### 2.4 Programming the Structures' Shape and Multi‐Stability Suppression

The frames' shape and multi‐stability can be tuned reversibly post‐fabrication. Taking a frame with the membrane in its melt state, it is possible to realize defined shapes by imposing boundary conditions/deformations to the strips during the cooling phase. Figure 4b illustrates the steps that can be followed to re‐program the structure's shape and three examples of new stable states realized by this method. While in its melt state, the frame is constrained by either forcing an arbitrary number of FRP strips to be flat or by realizing a saddle shape globally at room temperature. With this method, the multi‐stability is suppressed. To recover the equilibrium shape via the 1w‐SME, the membrane is re‐melted and allowed to cool down without external constraints. Different combinations of flat strips can be imposed on the frame; none of these states were achieved in previous studies.^[^ ^37^, ^38^ ^]^ Compared to a previous work, that demonstrated the possibility of switching between mono‐ and bi‐stable behavior by changing the pressure input of a “fluidic origami” structure,^[^ ^36^ ^]^ our approach exploits the reversible shape‐changing of shape memory polymers to realize new stables shape and suppression of the multi‐stability simultaneously.

This technique can be used also to impose a different magnitude of the deformations in the FRP strips while preserving the multi‐stability. To decrease the deformations of a frame in its stable state with four inflection points, the maximum height of the frame can be constrained by placing the structure during the cooling phase between two rigid plates kept at a fixed distance from one another. Figure 4c shows the frame with 25% pre‐stretching with an out‐of‐plane deformation of 8 mm in its equilibrium shape. The multi‐stability of the frame at room temperature is maintained for this case and its stable state with zero inflection points is illustrated in Figure 4c. In Section S12 (Supporting Information), we provide further experimental results demonstrating that with this technique any desired out‐of‐plane deformation lower than the one in the equilibrium shape can be realized. Experiments demonstrate that for this frame the multi‐stability is preserved if the out‐of‐plane deformations are higher than 6 mm. With a similar approach, is possible to increase the deformations of the strips at the user's needs, preserving the multi‐stability of the frame.

### 2.5 Periodic Meta‐Structures with Programmable Stable Shapes

**Figure 5:**
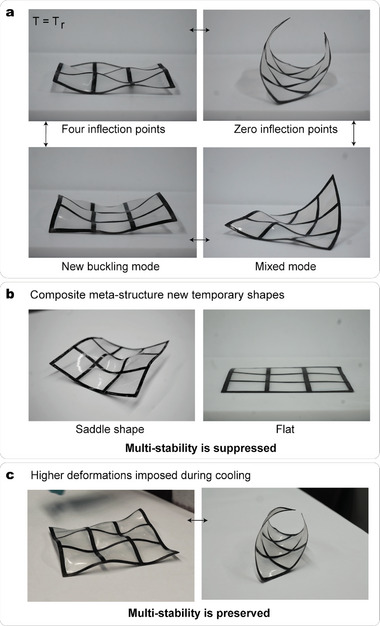
Periodic meta‐structures multi‐stability and tailorable deformations. a) The 3 × 3 grid is manufactured with each composite strip having a total length *L* = 140mm and width *w* = 5mm. The pre‐stretching of the membrane is ε=27%. The meta‐structure at room temperature is multi‐stable and has a large number of stable states. b) By imposing specific deformations during the cooling phase, new shapes are enabled. The periodic structure can realize a saddle locally (i.e., in the central cell of the grid) and can be made fully flat. c) By imposing higher deformations to the meta‐structures during the shape‐forming step, the shape of the meta‐structure can be tailored without suppression of the multi‐stability. The depicted configurations correspond from left to right to a stable state with zero and four inflection points.

We now investigate the behavior of periodic assemblies by manufacturing a 3 × 3 periodic meta‐structure with the same methods illustrated in the Experimental Section. **Figure** **5**a reports photographs of the structure after undergoing boundary‐free shape forming. The structure has the same multi‐stability as the meta‐structures introduced in previous work.^[^ ^38^ ^]^ The multi‐stability of the frame is preserved in periodic arrangements, with all strips having one, two, zero inflection points, or a mix of these. Moreover, the class of stable states with the new buckling mode is also realized. Recalling the result from previous studies,^[^ ^38^ ^]^ this class is characterized by strips of a unit cell possessing two inflection points. In Section S13 (Supporting Information), a more detailed description of the multi‐stability of the structures is provided.

Following the same experimental setup illustrated in Figure 4, the meta‐structure's deformations can be tailored to realize new stable shapes. Figure 5b shows two temporary shapes of the 3 × 3 grid where either the central cell is a saddle or all strips are flat. For these cases, the multi‐stability of the meta‐structure is temporarily suppressed and can be recovered upon heating to 60 °C, thanks to the 1w‐SME. Two examples of possible shapes that can be enforced with this method are shown. Combinations of unit cells in a saddle shape, flat, and with four inflection points are enabled, and examples of temporary shapes are presented in Section 2.6.

The deformations of the meta‐structure are temperature‐dependent and we can tune their shape and multi‐stability post‐fabrication. The magnitude of the deformations can be tailored at the user's will, though not exceeding the elastic range of the structure's sub‐constituents. In Figure 5c, an example of the grid in its state with all strips possessing zero inflection points, but much higher deformations than those of the boundary‐free structure is shown. This stable shape was obtained following the same procedure illustrated for the single frame in Figure 4b. Specifically, to fix this shape the structure is cooled down while keeping fixed the distance between the two opposite edges of the structure. In this case, the multi‐stability of the meta‐structure is preserved. Two examples of re‐programming of the meta‐structure's shape can be found in the Video S4 and Video S5 (Supporting Information). The video also reports the multi‐stability of the structures at room temperature. Overall, the behavior of the single cell is preserved in periodic assemblies.

### 2.6 Variable Stiffness, Actuation, and Optical Proprieties

**Figure 6:**
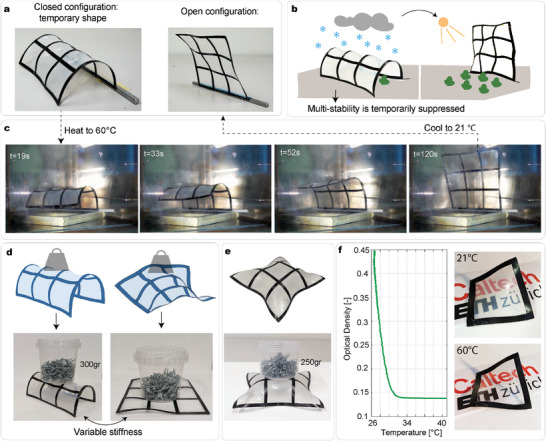
FRP meta‐structures possess a new temporary shape resembling a cylinder. a) The open and closed configuration of a meta‐structure. b) Schematic of an envisioned application: self‐sensing reconfigurable greenhouses. c) Shape transformation through a change in temperature of large structures is enabled. From the temporary cylindrical shape at room temperature, when exposed to T = 60 °C, the hybrid meta‐structure transitions between the cylindrical state to the boundary‐free one. The full video can be found in Video S6 (Supporting Information). d) Variable stiffness behavior of the structures. When the multi‐stability is temporarily suppressed, the structure can withstand a load that is 80 times its own weight. The same structure collapses under the same weight if it is in the boundary‐free shape formed stable state. e) FRP meta‐structures in another temporary shape resembling a pillow. By suppressing the multi‐stability and fixing the structure in this 3D shape, it can withstand an external load up to 250gr. f) Optical measurements. At the decrease in temperature, the optical density increases upon crystallization, and this can be visually seen by the naked eye.

The 3 × 3 periodic meta‐structure can be temporarily fixed to new stable states. By enforcing a cylindrical shape during the cool‐down, the stable shape illustrated in **Figure** **6**a is realized. The cylinder possesses constant curvature along one set of FRP strips parallel to one another. Video S5 (Supporting Information) illustrates the programming of this stable shape. The multi‐stability of the structure is temporarily suppressed, thus leading to an increase in the stiffness of the structure. To verify this assumption, a weight is placed on top of the structure in its cylindrical shape. Figure 6 shows that the grid, which alone weighs 3.75 gr, can withstand a weight of 300 gr when in its temporary cylindrical stable state. Taking the same structure in its boundary‐free shape (i.e., when it's multi‐stable), it collapses when subject to the same weight. Another example of a temporary shape is illustrated in Figure 6e. Here, the meta‐structure resembles a pillow and, in this shape, shows load‐bearing capability up to 250 gr. With this method, both the shape and stiffness response of the periodic structure can be tailored.

Moreover, the integration of an SMP membrane has the advantage of mitigating the complexity of actuating large meta‐structures. The hybrid structure achieves large transformations in response to a change in temperature. Figure 6c illustrates an example. The 3 × 3 structure in its cylindrical temporary shape is fixed along one edge to be kept in place. The grid is placed in an oven preheated to 60 °C. Snapshots from the shape change are reported in Figure 6c. In two minutes, the structure gradually deploys, moving to its fully open configuration due to the 1w‐SME. In the melt state, it is noted that the grid is slightly bent towards one side, due to gravity and manufacturing imperfections. Once the grid is cooled down to room temperature, the membrane crystallizes, going to the boundary‐free stable state illustrated in Figure 6a, labeled as “open configuration”. Due to the boundary conditions that are imposed on the grid along the edge fixed to the rod, it can be observed that the unit cells along the bottom edge possess a hybrid shape (with a combination of flat and buckled strips). The structure is shaped into a cylinder and actuated in the oven over five cycles showing the same behavior without structural damage. This demonstrates that the structure can overcome its own weight and transition to the stable state with four inflection points. Compared to other actuation methods proposed for other multi‐stable structures,^[^ ^27^, ^36^ ^]^ our meta‐structures show a slow reconfiguration speed that ensures a controlled change of shape. The speed is limited by the rapidity of heat transfer to the SMP, as well as by the thermal and mechanical properties of the SMP. These limitations pose challenges for the use of our structures in applications where a rapid snap‐through shape change is desirable (e.g., jumping robots, fast grippers).

To the naked eye, the membrane appears transparent in the melt state, while it turns opaque when the polymer crystallizes. This phenomenon is highlighted in Figure 6f, where the same frame is placed over the ETH and Caltech logos at 21 and 60 °C. Given that the behavior of hybrid FRP meta‐structures varies with temperature, monitoring changes in the opacity of the membrane could potentially serve as a useful optical readout providing information about both the temperature and level of crystallization. To test this hypothesis, optical measurements are performed by heating a membrane sample affixed to a glass slide with a heat gun to approximately 60 °C and then placing it into a UV‐visible spectrometer while monitoring the optical density at a single wavelength λ = 400 nm as it cools to room temperature. Results are illustrated in Figure 6f and show that the optical density increases significantly as the temperature decreases, consistent with an increase in light scattering due to the formation of crystalline domains within the membrane. Constant temperature spectra at *T*_*r*_ as a function of wavelength show a characteristic rise as the wavelength decreases towards the ultraviolet region (See Figure S19, Supporting Information) that is consistent with light scattering as observed in semi‐crystalline polypropylene in a study that also modeled such data utilizing an approach based on Mie scattering theory.^[^ ^45^ ^]^ While modeling the kinetic data is beyond the scope of the current study, the experiments reported here suggest it would be possible to precisely monitor both the temperature and the degree of crystallization of the membrane in a real‐world application via optical sensing.

Being able to withstand loads of about 80 times their weight, hybrid composite meta‐structures show potential for applications where load‐carrying capability is a requirement. With the possibility of realizing shape changes in response to a change in temperature, we envision adaptive greenhouses as a potential application (see Figure 6b). Within this application, the structures are exposed to different weather conditions (such as rain or snow) that would result in high loads. Future studies can be conducted to foster the properties of hybrid meta‐structure to realize greenhouses that, after a winter season, self‐reconfigure into their open configuration. The proposed actuation allows transformation from the cylindrical state to the open one automatically. However, we note that an external actuation should be developed to reverse the shape change. The use of pneumatic actuators^[^ ^38^ ^]^ or some other means of accomplishing the mechanical work associated with reprogramming could be considered. Further, it is worth emphasizing that a reversible design concept could be developed in the future that utilizes the reversible changes observed in unit cell deformation resulting from temperature‐dependent effects on internal stresses arising from the 2w‐SME (See Figure 3) as the means for actuation. Toward this application, the change in opacity observed in the membranes when switching between the melted and crystallized states can be exploited to enhance the structure's functionalities, enabling tuning of the amount of light transmitted into the greenhouse, as well as optical monitoring of the temperature of the membrane.

## 3 Discussion

We present a programmable multi‐stable meta‐structure that combines flat, thin FRP shells with bi‐axially pre‐stretched, cross‐linked PCOE membranes. The interplay between the stress mismatch between the two constituents, the 1w‐SME and 2w‐SME, and temperature‐dependent material properties pushes the boundaries of current multi‐stable elements, opening new features in the design of adaptive structures. The concept allows the realization of multi‐stable meta‐structures whose shape and multi‐stability can be reversibly tuned post‐fabrication. In contrast with other works where the 2w‐SME was observed for polymers subject to uni‐axial stresses only, this study demonstrates, for the first time, that the 2w‐SME is enabled by bi‐axial stress under constant strain conditions. Taking advantage of this effect, as well as the 1w‐SME, the multi‐stability of the meta‐structure can be turned on and off at the user's will, the magnitude of deformations in the structure is tunable, and new stable shapes are realized. Commonly, SMPs can memorize the permanent shape and recover it via the 1w‐SME from a temporary shape when an external stimulus is applied.^[^ ^46^, ^47^ ^]^ The presented design combines this ability with multi‐stability, enlarging the range of achievable shapes.

Programming the multi‐stability is enabled by applying a temperature gradient to melt the SMP and, thereafter, by imposing a desired 3D shape to the meta‐structure during cooling. Experimental results show that the programming process is repeatable and offers the possibility for self‐actuation. With this concept, the actuation of large FRP structures can be partially performed with temperature, reducing the need for external actuators previously developed,^[^ ^38^ ^]^ decreasing the structures' weight, and reducing manufacturing/assembling complexity. Moreover, the stiffness of FRP meta‐structures can be tuned post‐fabrication. The structures do not possess load‐carrying capability when the membrane is in its melt state but if fixed in specific shapes that can well distribute external weights (such as a cylinder), the structure can withstand up to 80 times its weight when at room temperature. Importantly, the temporary suppression of the multi‐stability of the structures drastically reduces the risk of undesired snapping when the structures are perturbed by some external force, and avoids marginal stability; an important design constraint when integrating multi‐stable elements in adaptive systems.

The research presented herein utilizes a PCOE shape memory polymer cross‐linked with dibenzoyl peroxide with a 2.5 wt.% loading. By tuning the loading of the dibenzoyl peroxide, it is possible to shift the crystallization and melting peaks of the polymer (See Figure S10, Supporting Information). In the Table S3 (Supporting Information) reports the results from DSC performed on samples with a wt.∈[0.0,..,3.5]%. The results show that *T*_*m*_ can vary between 56.8 and 46.6 °C within this range. Moreover, by substituting the dibenzoyl peroxide with dicumyl peroxide it is possible to shift the *T*_*m*_ and *T*_*c*_ of the polymer by adjusting the cross‐linker loading from *T*_*m*_ = 48  °C for 1 wt.%, to below room temperature for higher loadings as has been observed herein (See Figure S9 and Table S2, Supporting Information and Ref. [42]). Controlling cross‐linker loading can be used to tune the transition temperature at which the shape memory effect occurs, broadening the range of potential applications. This feature could be exploited to change the temperatures at which desired changes of configuration occur or, for example, to realize wearable devices that self‐shape themselves to the human body when exposed to the skin (i.e., *T*_*m*_ below 37 °C).

Being able to re‐program the energy landscape of FRP meta‐structures offers the possibility to augment the functionality of existing multi‐stable concepts, showing potential for the realization of variable stiffness structures. To better understand the role of the shape memory effect in the multi‐stability behavior of the structure, the FE model developed in previous works for the TPU membrane^[^ ^38^ ^]^ should be expanded to capture the temperature‐dependent properties of the shape memory polymer. To realize this model, novel techniques that can capture the two‐way shape memory effect are needed. To the authors' knowledge current material models that describe one‐way shape memory polymers are unable to predict the properties of our architecture.^[^ ^48^, ^49^ ^]^ Modeling of hybrid frames will be the subject of future studies and the model will serve as a powerful resource to fully characterize the energy landscape of the proposed structures.^[^ ^50^ ^]^ Moreover, to explore the use of this concept for future applications, new structures with other geometries in aperiodic assemblies, different loading of the cross‐linker, and varying the pre‐stretching of the membrane in periodic structures could be investigated.

## 4 Experimental Section

### SMP Molding into a Thin Membrane and Pre‐Stretching

To mold the polymer chunks into a thin membrane of uniform thickness the Fontjine hot press was employed. Polymer chunks containing cross‐linker were then sliced into thin pieces (1mm thickness) and compressed into flat membranes and cross‐linked using a vacuum hot press. For this step, two steel plates (20mm thickness, 300x17mm area) were coated with a thin Kapton foil, and the polymer chunks were placed between them. Two steel spacers were placed between the steel plates to control the thickness. The sample was then placed in the Fontjine hot press with a vacuum on. The sample was first heated to 60 °C, then a pressure of eight bars was applied for 15min. Afterward, the temperature was brought to 140 °C, and the sample was left to cure for 12h. Figure S1 (Supporting Information) illustrates the two steel plates and the polymer before and after the curing. Once manufactured, the membrane was cooled to room temperature and removed from the plates. Attempts to shape the cross‐linked membrane by manually pressing the membrane with C‐clamps were observed to be of similar character, although with a poorly uniform thickness.

### Assembling of the Frames and Grids

To assemble the frame, the membrane was heated up to melting temperature using a heat gun adjusted to a low heat setting (approx 60 °C). In the melt state, the membrane was bi‐axially pre‐stretched with a pre‐stretching ε_*x*_ = ε_*y*_ = ε. To hold the membrane in its pre‐stressed state white cooling, a large steel frame, and some magnets were employed (as in previous studies^[^ ^37^ ^]^). A unit cell consisted of four identical rectangular strips manufactured employing HR40/513 epoxy prepreg from NTPT and a [0/90/0] layup. Details about the material were given in the Supporting Information. The strips were connected with each other in a frame‐like square structure with overlapping corners resembling a plain‐weave pattern, the same material, layup, and weave pattern were followed to manufacture the periodic grids of this study. The bonding between the two constituents was performed in the flat configuration (x‐y plane) employing Loctite plastics superglue. The shape‐forming mechanism arose when heat was applied using a heat gun adjusted to a low heat setting (approx 60 °C).

### Membrane Material Characterization

Details about membrane synthesis, material characterization, and testing can be found in the Supporting Information.

## Conflict of Interest

The authors declare no conflict of interest.

## Author Contributions

G. Risso and M. Kudisch equally contributed to the work. G. Risso and M. Kudisch conceived the project, and performed all theoretical development, and experiments. P. Ermanni and C. Daraio provided guidance throughout the research, administrated the project, and acquired the funding. G. Risso and M. Kudisch wrote the manuscript with inputs from all authors.

## Supporting information

Supporting Information

Supporting Video S2

Supporting Video S3

Supporting Video_S4

Supporting Video S5

Supporting Video S6

## Data Availability

The data that support the findings of this study are available in the supplementary material of this article.
