# Supplementary material for: Tuning the Properties of Multi‐Stable Structures Post‐Fabrication Via the Two‐Way Shape Memory Polymer Effect
Source: Adv Sci (Weinh). 2024 Mar 17;11(21):2308903. doi: 10.1002/advs.202308903 (PMC11151059; doi:10.1002/advs.202308903)
Supplement: Supplementary file 1 — Supporting Information [file ADVS-11-2308903-s005.pdf]

ADVANCED SCIENCE Open Access

Supporting Information

for *Adv. Sci.*, DOI 10.1002/advs.202308903

Tuning the Properties of Multi-Stable Structures Post-Fabrication Via the Two-Way Shape Memory Polymer Effect

Giada Risso, Max Kudisch, Paolo Ermanni and Chiara Daraio*

Supporting Information

For

Post-fabrication tuning of the properties of multi-stable structures via two-way shape memory polymer effect

Giada Risso, Max Kudisch, Paolo Ermanni, Chiara Daraio

Table of Contents

1. Supporting Figures 2

2. Membrane Fabrication 3

3. General Reagent Information 3

4. Repeatability study 4

5. Differential Scanning Calorimetry 4

6. Dynamic Mechanical Analysis 11

7. UV-visible Spectroscopy 17

8. Uniaxial Tensile Testing 21

9. Membrane Raw Material Synthesis 24

10. Actuation Work Testing 25

11. Composite Material Details 26

12. Tailoring the out-of-plane deformation of FRP frames 27

13. Description of the multi-stability of periodic structures 27

14. Bibliography 29

1. Supporting Figures

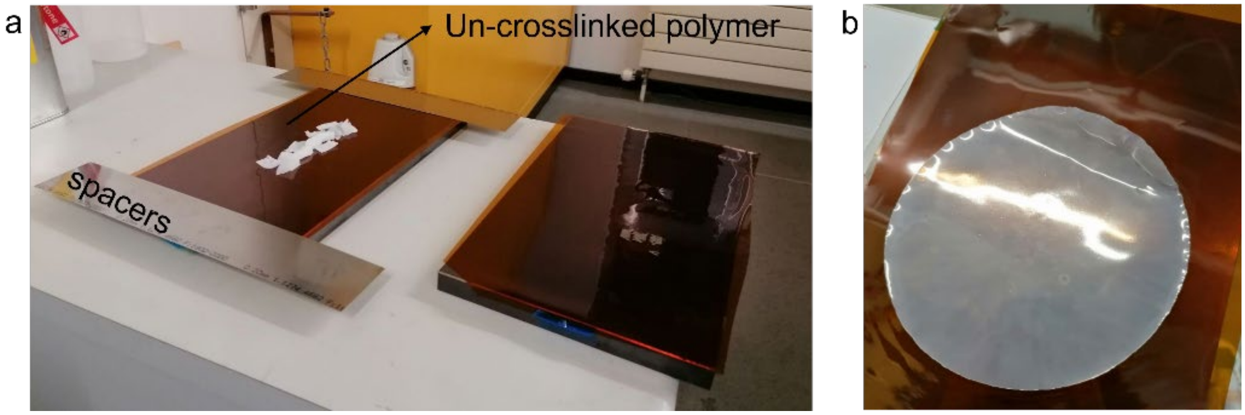

Figure S1: Molding of the polymer into a thin membrane. **a)** The polymer chunks are placed on the steel plate coated with a Kapton foil. Two spacers, placed between the plates, ensure thickness control. **b)** An example of membrane, lying on a steel plate, after completed crosslinking process.

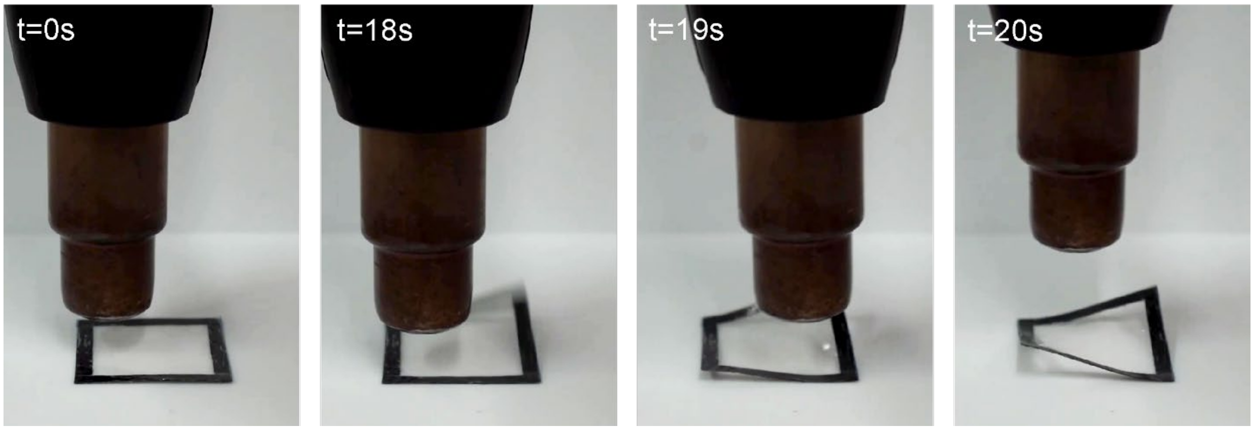

Figure S2: Snapshots from boundary free shape-forming phenomenon of a frame with length L=50 mm, width w=5 mm, and pre-stretching 25%. The heat gun is set to 60 °C. The snapshots are taken from the supplementary video 1.

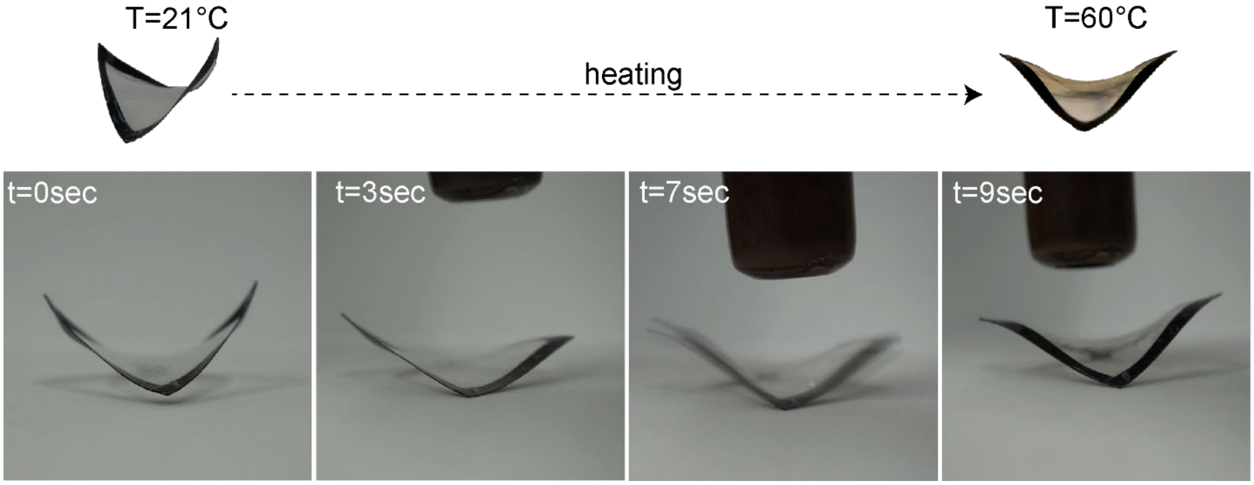

Figure S3: Actuation by temperature-induced shape change with snapshots going from zero inflection points at room temperature to four inflection points at 60 °C. For this test, one corner of the frame is bonded to the ground to fix the structure in place. The snapshots are taken from the supplementary video 2.

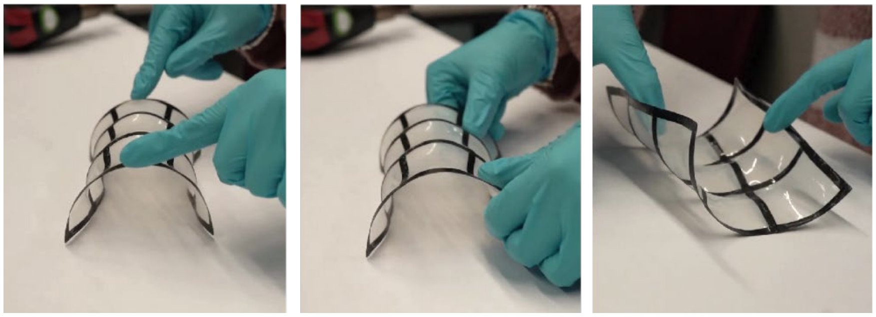

Figure S4: Cylindrical temporary shape: the multi-stability is suppressed.

2. Membrane Fabrication

Crosslinked poly(*cis*-cyclooctene) (PCOE) was fabricated into a membrane form by loading chunks of polymer onto a steel plate as shown in Figure S1. The steel plate was inserted into a vacuum hot press (Fontijne) and crosslinked under pressure (8 bar) at a temperature of 140° C for 12h hours, with vacuum. The steel plates setup is reported in Figure S1a.

3. General Reagent Information

Toluene (>99.5%, ACS Grade, Aldrich), dibenzoyl peroxide (>75%, wetted with ca. 25% H_2O, stored at 5°C, TCI), dicumyl peroxide (99% purity, Acros Organics), and poly(*cis*-cyclooctene) (Vestenamer 8012, Evonik) were stored as recommended by the manufacturer and used as received.

4. Repeatability study

To experimentally validate that the change in out-of-plane deformation of the frames with a temperature gradient is reversible and repeatable over time, we performed a cyclic repeatability test (of 10 iterations) on the frame with pre-stretching $\varepsilon = 10\%$.

First the maximum out-of-plane deformation, $\boldsymbol{z_{max}}$, of the frame that is at room temperature is calculated using a ruler frame. Second, the frame is heated up to 60 °C. While in the melt state, $\boldsymbol{z_{max}}$ is calculated. Afterward, the frame is let to cool down to room temperature for at least 3h. Upon cooling, $\boldsymbol{z_{max}}$ is calculated and a new heating/cooling cycle is started. Table S1 reports the results at each # cycle. The process is highly repeatable. $\boldsymbol{z_{max}}$ shows a variation of ±1mm, which is the precision of the measurement tool employed for this experiment.

#	$\boldsymbol{z_{max}}$ at **21 °C** [mm]	$\boldsymbol{z_{max}}$ at **60 °C** [mm]
1	8	3
2	10	4
3	10	4
4	9	3
5	10	4
6	10	4
7	9	5
8	10	4
9	10	4
10	9	4

Table S1: Cyclic test performed over a frame with length L=50 mm, width w=5 mm, and pre-stretching 10%.

5. Differential Scanning Calorimetry

General Procedure.

Differential Scanning Calorimetry (DSC) was performed utilizing a TA Instruments DSC 25, which was calibrated prior to use according to the manufacturer recommendations with an indium standard (TA Instruments). Samples (for synthesis, see Section 6) were cut to fit into a Tzero aluminum pan which was hermetically sealed under air. Air was used as the purge gas at a flow rate of 50 mL/min for all experiments. After placing the hermetically sealed pan into the instrument with an empty hermetically sealed reference pan, the run was programmed according to the following standard profile.

1. Equilibrate at 30 °C
2. Ramp to 100 °C at 10 °C/min
3. Ramp from 100 °C to -40 °C at 10 °C/min
4. Ramp from -40 °C to 100 °C at 10 °C/min
5. Ramp from 100 °C to -40 °C at 10 °C/min

The first cycle (1-3) was utilized to standardize the thermal history of each sample, while the second cycle (4-5) is reported.

Membrane Characterization.

A membrane sample representative of that used to prepare all structures was made as described in Figure S1 and a small piece was cut and removed for DSC analysis.

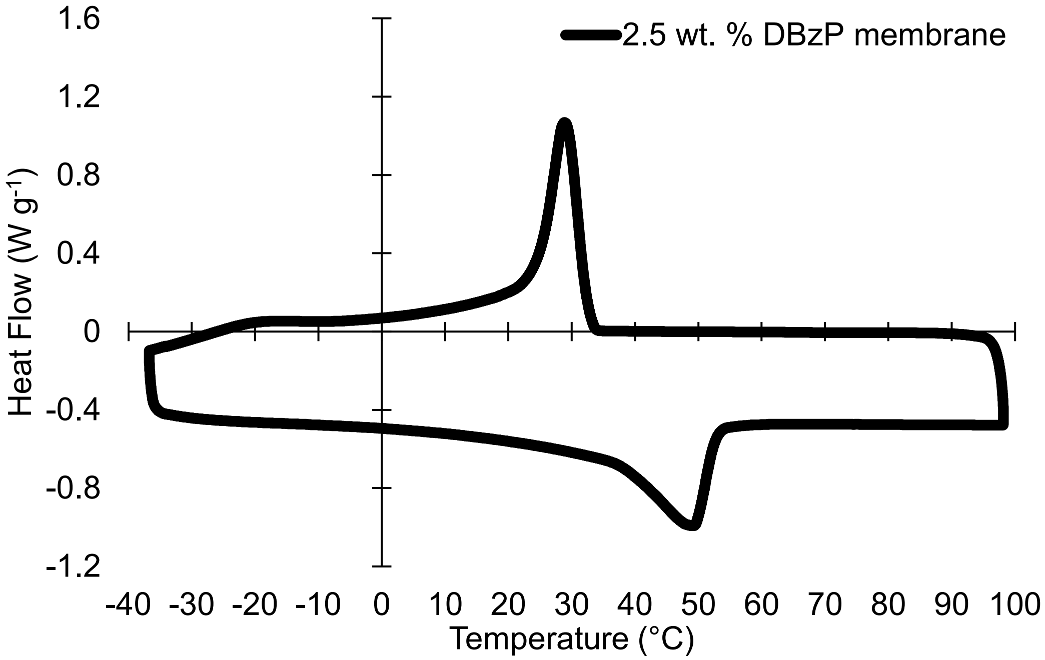

Figure S5: 2nd cycle DSC heating and cooling curves for membrane sample representative of composition used to make structures (i.e. 2.5 wt. % DBzP crosslinked in a vacuum hot press). Exothermic peaks pointing up. T_m = 49.17 °C and T_c = 28.84 °C. Normalized enthalpy of melting = 57.2 J g^{-1}.

Kinetics of crosslinking with DBzP.

In previous work by one of our groups,[1] samples were created and analyzed by DSC to follow the kinetics of cross-linking with DBzP. The following method description is reproduced with modification from that work for convenience of the reader.

~10 mg of PCOE containing DBzP (2.5 wt. %) prepared as described in the Materials Synthesis section below was placed on a circular glass coverslip and sandwiched between 2 PTFE plates, which were in turn sandwiched between 2 steel plates. The assembly was heated to 70 °C in an oven to melt the PCOE and then compressed with C clamps (4x 1.5 in and 2x 4.0 in). The compressed assembly was placed in a vacuum oven (VWR Symphony) and crosslinked under vacuum at 140 °C for varying amounts of time (1, 2, 4, or 24 h). A small piece (approx. 1-3 mg) was removed from each crosslinked sample for DSC analysis.

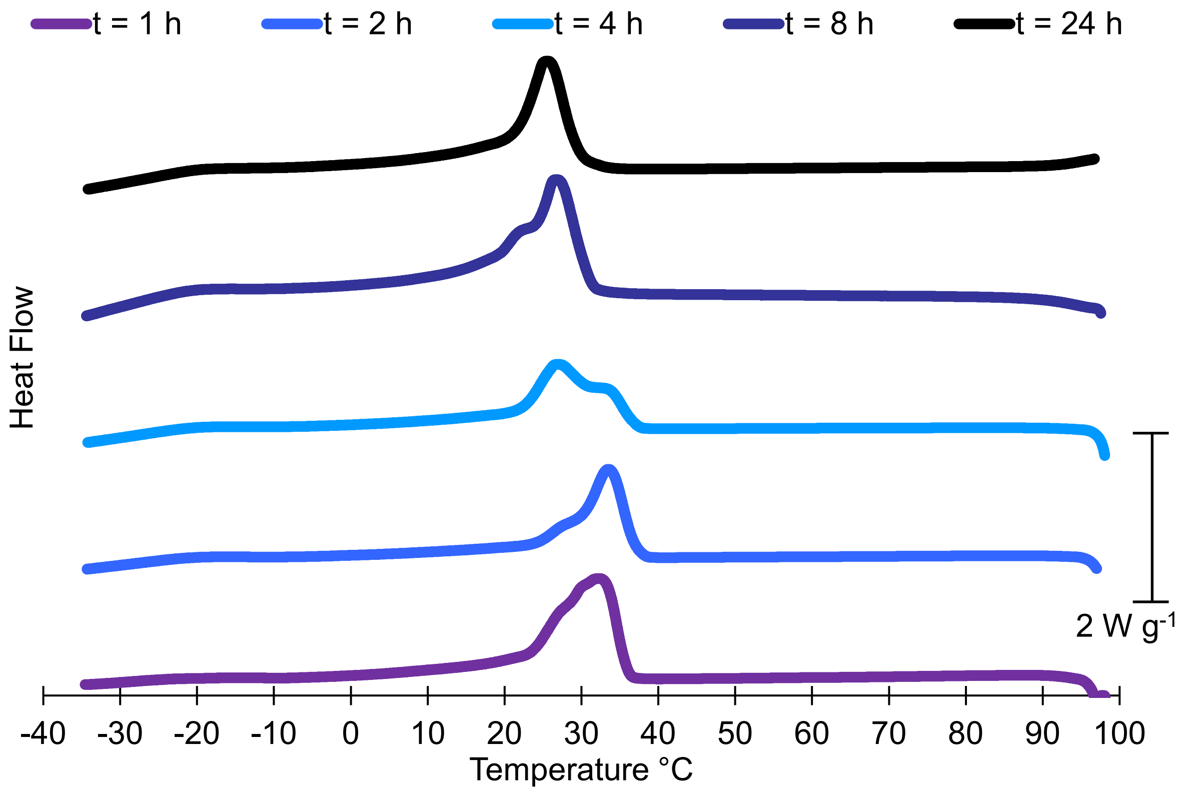

Figure S6: DSC cooling curves for PCOE membrane samples (2.5 wt. % DBzP) crosslinked at 140 °C for increasing amounts of time (1-24 h). Exothermic peaks pointing up. This plot is reproduced (modified with offset traces for improved clarity) from one of the authors' previous works.[1]

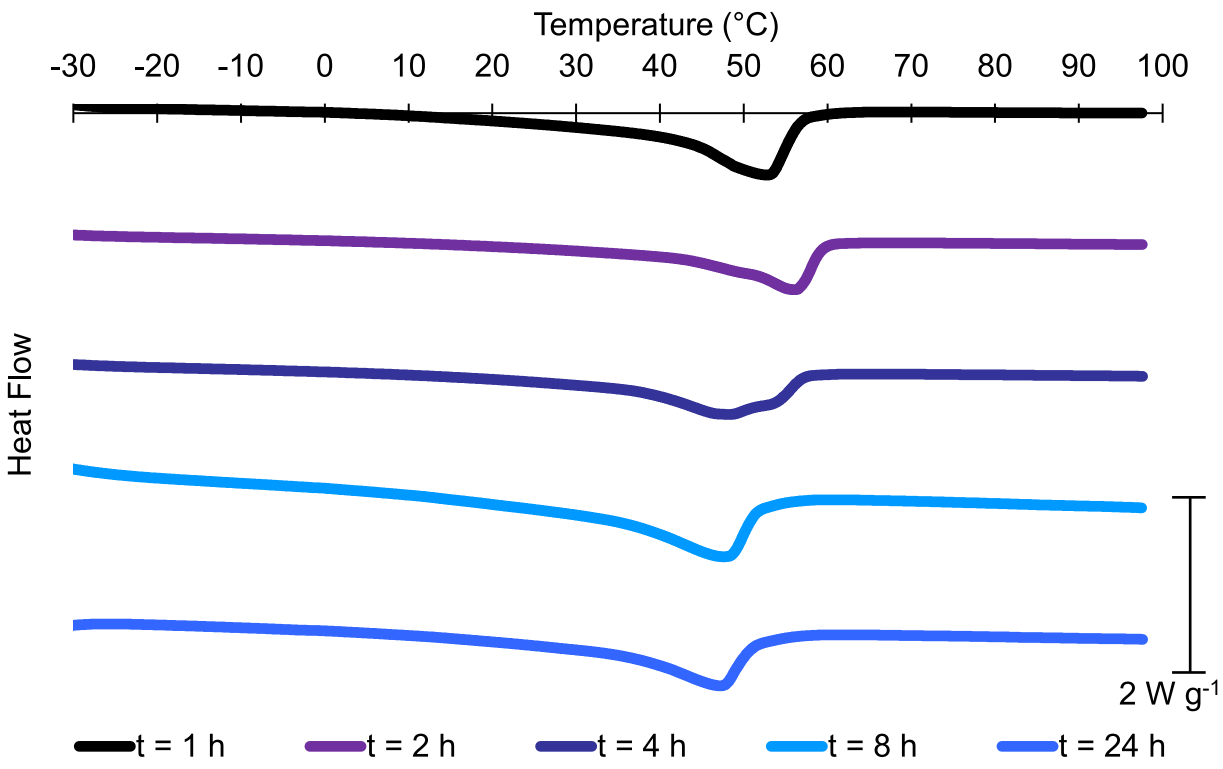

Figure S7: DSC heating curves for PCOE membrane samples (2.5 wt. % DBzP) crosslinked at 140 °C for increasing amounts of time (1-24 h). Exothermic peaks pointing up. This plot is reproduced (modified with offset traces for improved clarity) from one of the authors' previous works.[1]

While bimodal crystallization or melting curves are initially observed in the thermograms at short crosslinking times, the curves evolve to a single peak at longer times. The bimodal nature can be attributed to the presence of distinct populations of crosslinked and uncrosslinked PCOE crystallites resulting from partial conversion in the crosslinking reaction. As the reaction reaches higher conversion, a single population is obtained with melting or crystallization temperature consistent with the crosslinker loading (2.5 wt. % DBzP).

T_m decreases linearly with crosslinker loading.

In previous work by one of our groups,[1] samples were created and analyzed by DSC to test the hypothesis that increasing crosslinker loading would result in a decrease in T_m. The following method description is reproduced with modification from that work for convenience of the reader.

A series of 4 samples were synthesized with increasing dicumyl peroxide (DCP) loading (0-2%). Cylindrical molded samples were prepared as described in the Membrane Materials Synthesis Section. A small piece (3-15 mg) was removed for DSC analysis.

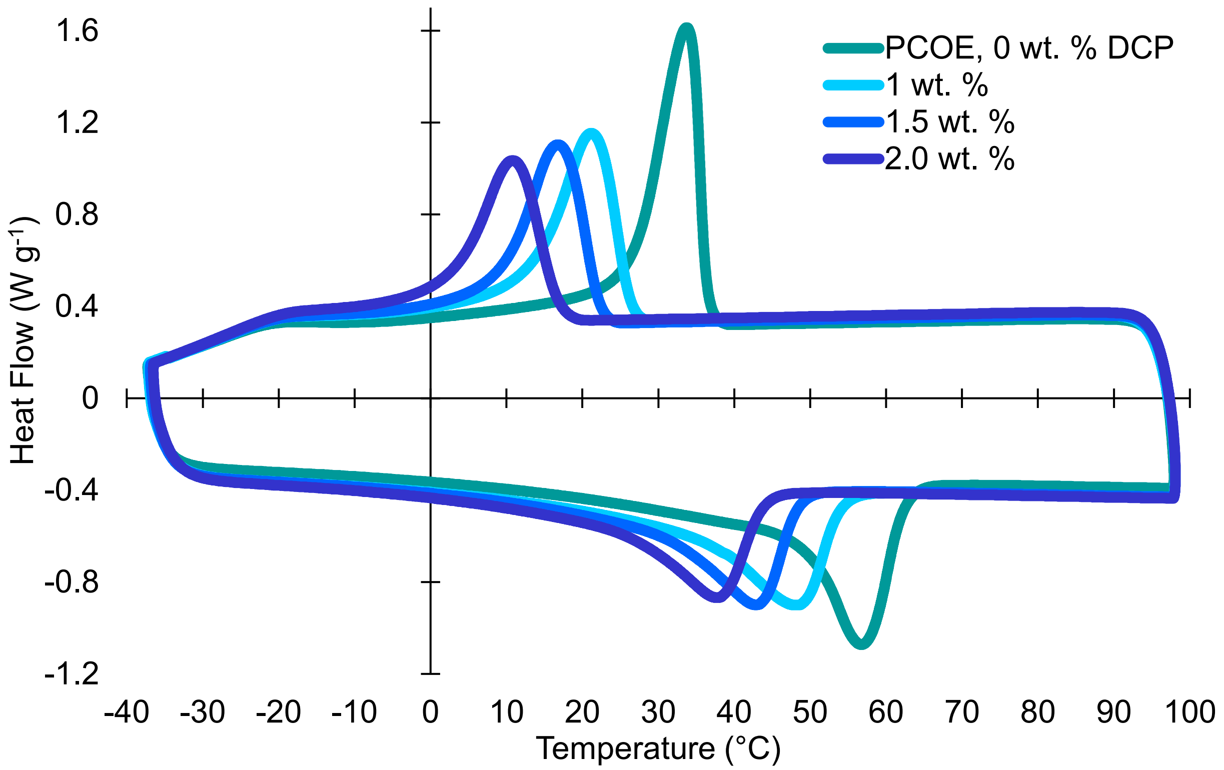

Figure S8: DSC thermograms for starting PCOE and after crosslinking with 1.0-2.0 wt. % dicumyl peroxide. Exothermic peaks pointing up.

Table S2: Parameters from DSC. T_m and T_c are obtained from the minimum or maximum of the melting or crystallization peak in DSC data above, respectively. Enthalpy was obtained by integration of each melting curve over a range of -20.0 to 70.0 °C. [a]Data in these columns is reproduced from previous work by one of our groups.[1] [b]Degree of crystallinity obtained using a literature value of ΔH = 216 J g^{-1}.[2]

DCP Loading, wt. %	T_m (°C)[a]	T_c (°C)[a]	ΔH (J g^{-1})	X_c[b]
uncrosslinked	56.8	33.6	76.0	0.35
1.0	48.2	20.9	64.1	0.30
1.5	42.9	16.8	58.9	0.27
2.0	37.7	10.9	53.6	0.25

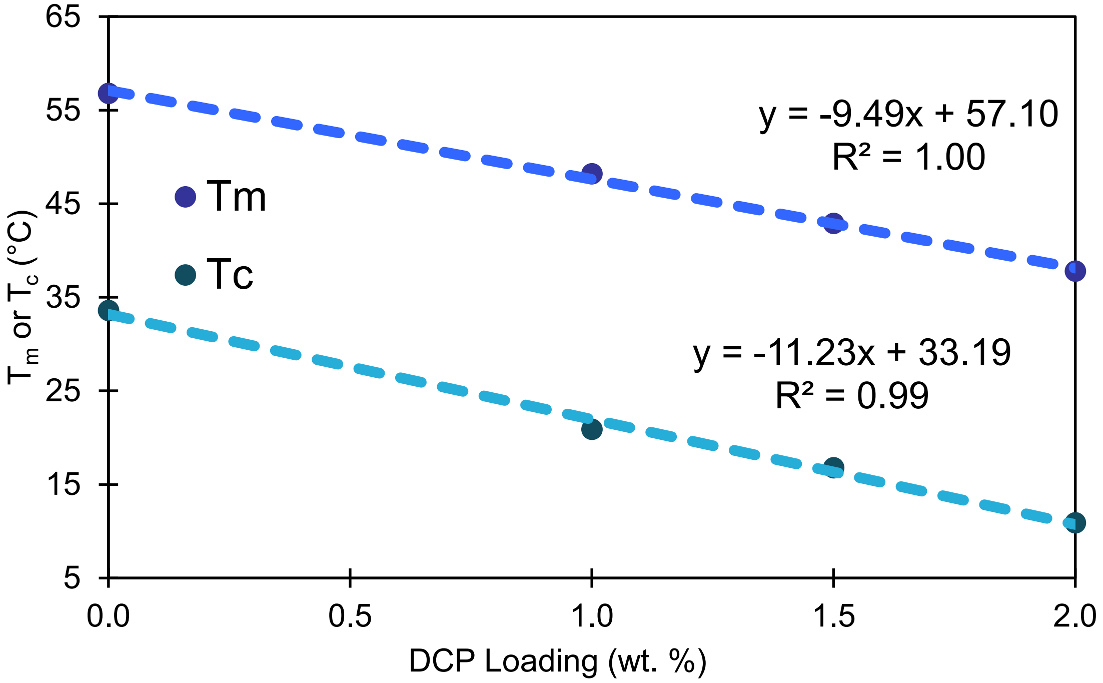

Figure S9: Melt transition (T_m) and crystallization (T_c) temperatures as a function of crosslinker dicumyl peroxide (DCP) loading. T_m is obtained from the minima and maxima, respectively, of DSC curves. Linear fits are shown. This plot is reproduced (with modification) from one of the authors' previous works.[1]

In previous work by one of our groups,[1] samples were created and analyzed by DSC to test the hypothesis that increasing dibenzoyl peroxide loading would result in a decrease in T_m. The following method description is reproduced with modification from that work for convenience of the reader.

A series of 8 samples were synthesized with increasing dibenzoyl peroxide loading (0.5-4.0 wt. %, relative to polymer). We note that these cylindrical form samples also contained 1 wt. % of hollow glass microspheres (K25, manufacturer 3M). This additive at such low loading has been shown to have no significant effect on the thermal properties of the cross-linked polymer (See T_m in Fig. 25A in referenced work).[1]

Table S3: Parameters from DSC. T_m and T_c are obtained from the minimum or maximum of the melting or crystallization peak in DSC data above, respectively. Enthalpy was obtained by integration of each melting curve over a range of -20.0 to 70.0 °C. [a]Data in these columns is reproduced from from previous work by one of our groups.[1] [b]Degree of crystallinity obtained using a literature value of ΔH = 216 J g^{-1}.[2]

DBzP Loading, wt. %	T_m (°C)[a]	T_c (°C)[a]	ΔH (J g^{-1})	X_c[b]
uncrosslinked	56.8	33.6	76.0	0.35
0.5	56.6	31.4	66.3	0.31
1.0	55.0	30.5	69.1	0.32
1.5	53.6	26.6	67.0	0.31
2.0	54.0	25.0	63.7	0.29
2.5	49.1	21.9	60.2	0.28
3.0	46.4	18.9	57.8	0.27
3.5	46.6	18.5	57.9	0.27
4.0	44.4	18.0	56.7	0.26

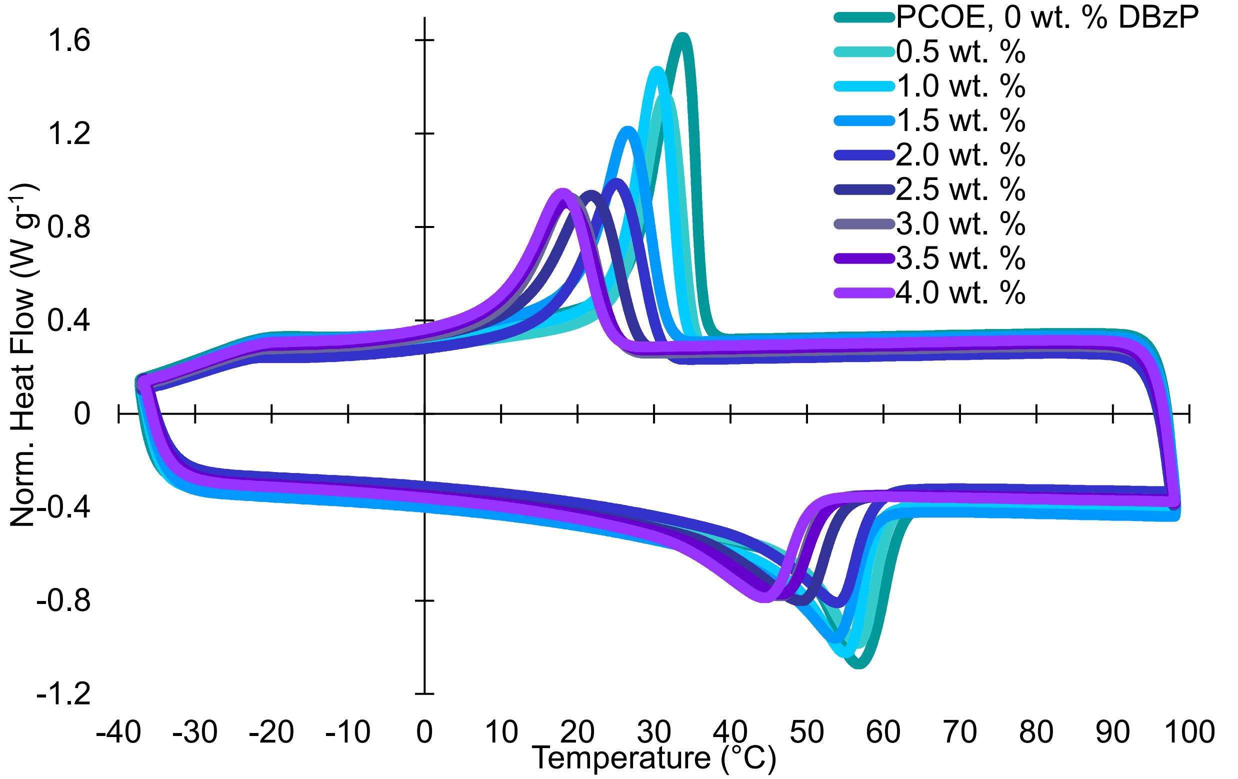

Figure S10: DSC thermograms for starting PCOE and after crosslinking with 0.5-4.0 wt. % dibenzoyl peroxide. Exothermic peaks pointing up.

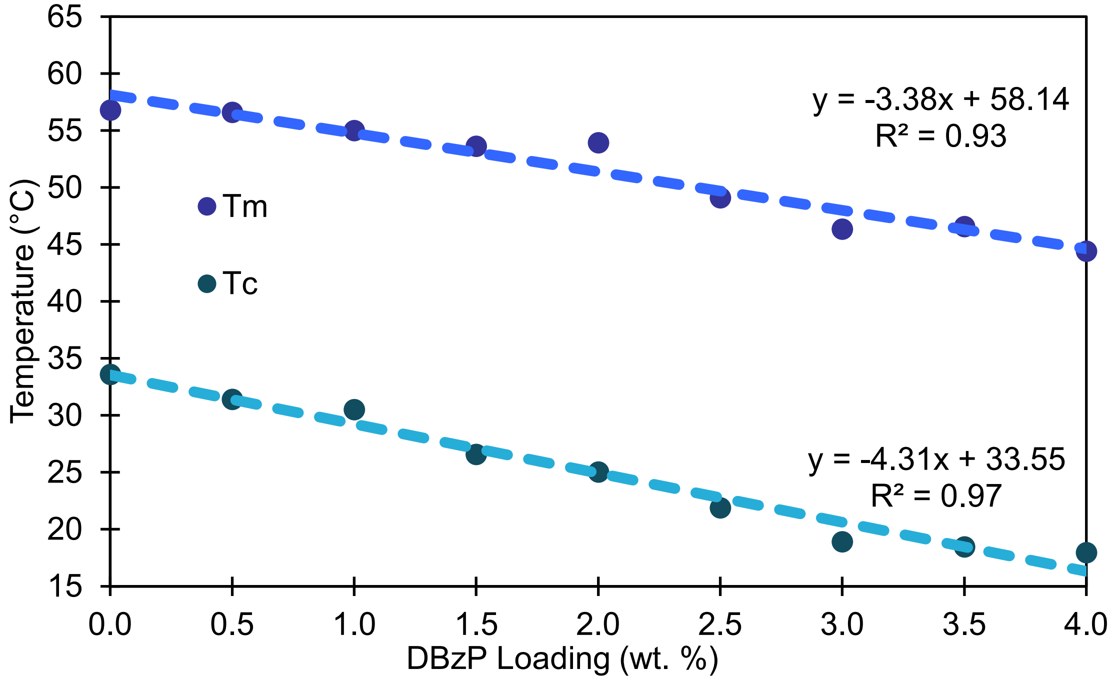

Figure S11: Melt transition (T_m) and crystallization (T_c) temperatures as a function of crosslinker dibenzoyl peroxide (DBzP) loading. T_m and T_c are obtained from the minima and maxima, respectively, of DSC curves shown above. Linear fits are shown. This plot is reproduced (with modification) from previous work by one of our groups.[1]

The lower R^2 values and greater variation in the data are most likely because DBzP is shipped stabilized with ~25 wt. % H_2O as a stabilizer. The wetted powder therefore varies in water content due to equilibrium with moisture in the air, resulting in variability when weighing out the crosslinker. DCP, on the other hand, does not require a stabilizer due to its increased stability at room temperature.

DCP Compared with DBzP as Crosslinker.

Both crosslinkers can be used to effectively synthesize membranes with suitable thermal and mechanical properties to construct reprogrammable, adaptive structures. DCP affords improved reproducibility since it does not require H_2O as a stabilizer. However, it requires a significantly higher crosslinking temperature to activate as compared with DBzP.

6. Dynamic Mechanical Analysis

Dynamic Mechanical Analysis (DMA) was performed with a DMA Q850 (TA instruments) that was calibrated according to the manufacturer's instructions prior to each experiment using a steel shim. Experiments were performed in tension using the uniaxial dual screw thin film clamp setup (TA instruments). Liquid nitrogen was utilized for cooling stages.

Storage and Loss Moduli from -145 to 100 °C

The following programming was used to measure storage and loss moduli.

1. Preload with force = 0.01 N
2. Oscillating strain sweep
3. Oscillating ramp from room temperature to 100 °C at 5 °C/min
4. Oscillating ramp from 100 °C to -145 °C at 5 °C/min
5. Oscillating ramp from -145 °C to 100 °C at 2 °C/min

The sample used for testing had an initial clamped length of 6.46475 mm, width of 5.11 mm, and thickness of 0.188 mm. A frequency of 1 Hz was utilized for oscillations. The oscillation stress reached a maximum of 1.98 MPa at -145.53 °C. The oscillating strain remained below 0.07% throughout steps 2-5.

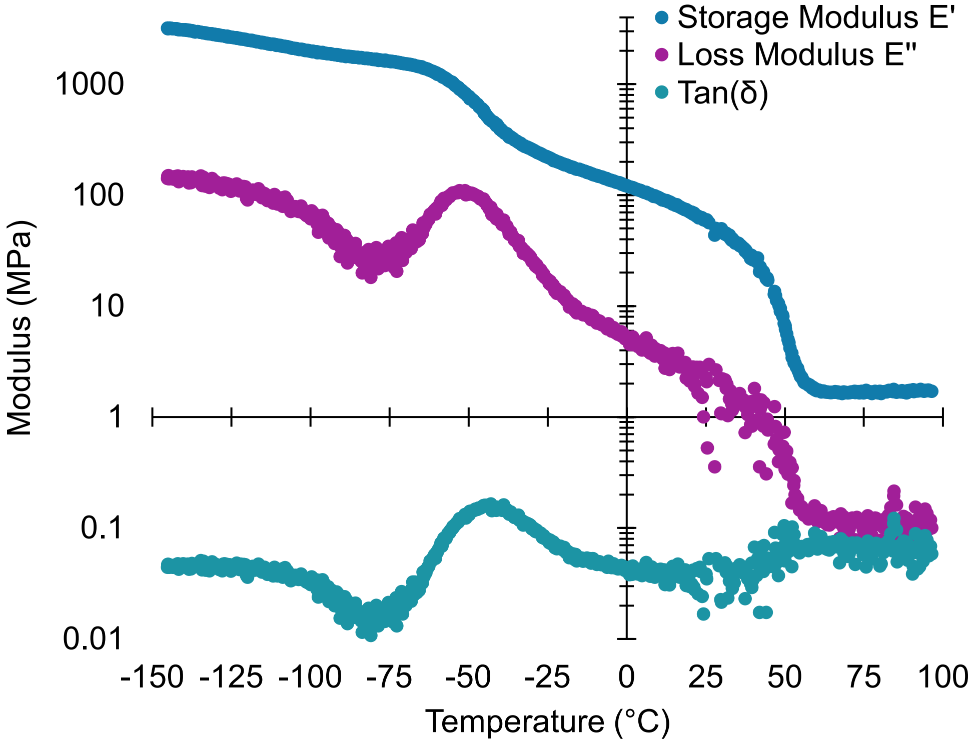

***Figure S12**:* Storage/loss moduli and tan(δ) for PCOE membrane sample prepared as in Fig. S1 upon heating from -145 to 100 °C.

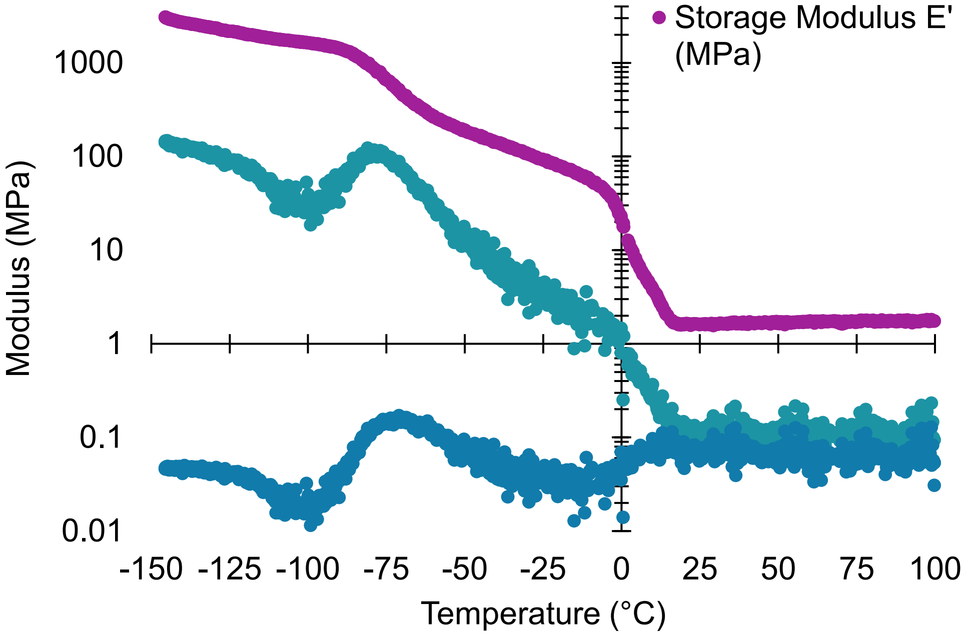

Figure S13: Storage/loss moduli and tan(δ) for PCOE membrane sample prepared as in *Fig. S1* upon cooling from 100 to -145 °C.

Thermomechanical Testing

The following program was used for thermomechanical tests, where X% strain = 10%, 15%, 20%, 25%, or 30%.

1. Equilibrate temperature at 60 °C for 5 min.
2. Strain ramp from 0% strain to X% strain at 1.0%/min.
3. Maintain strain for 1 min.
4. Equilibrate temperature at 55 °C for 0 min.
5. Equilibrate temperature at 45 °C for 0 min.
6. Equilibrate temperature at 35 °C for 0 min.
7. Equilibrate temperature at 25 °C for 0 min.
8. Equilibrate temperature at 15 °C for 0 min.
9. Equilibrate temperature at 5 °C for 0 min.
10. Equilibrate temperature at -10 °C for 10 min.
11. Equilibrate temperature at 0 °C for 0 min.
12. Equilibrate temperature at 10 °C for 0 min.
13. Equilibrate temperature at 20 °C for 0 min.
14. Equilibrate temperature at 30 °C for 0 min.
15. Equilibrate temperature at 40 °C for 0 min.

16. Equilibrate temperature at 50 °C for 0 min.
17. Equilibrate temperature at 60 °C for 0 min.
18. Equilibrate temperature at 70 °C for 0 min.
19. Equilibrate temperature at 80 °C for 0 min.
20. Equilibrate temperature at 90 °C for 0 min.
21. Equilibrate temperature at 100 °C for 0 min.

Stepwise equilibration was used to control the rate of temperature ramping since the DMA did not allow for programming ramping without oscillations while maintaining constant strain. The average ramp rate per equilibration segment ranged from 1.5 – 4.0 °C/min.

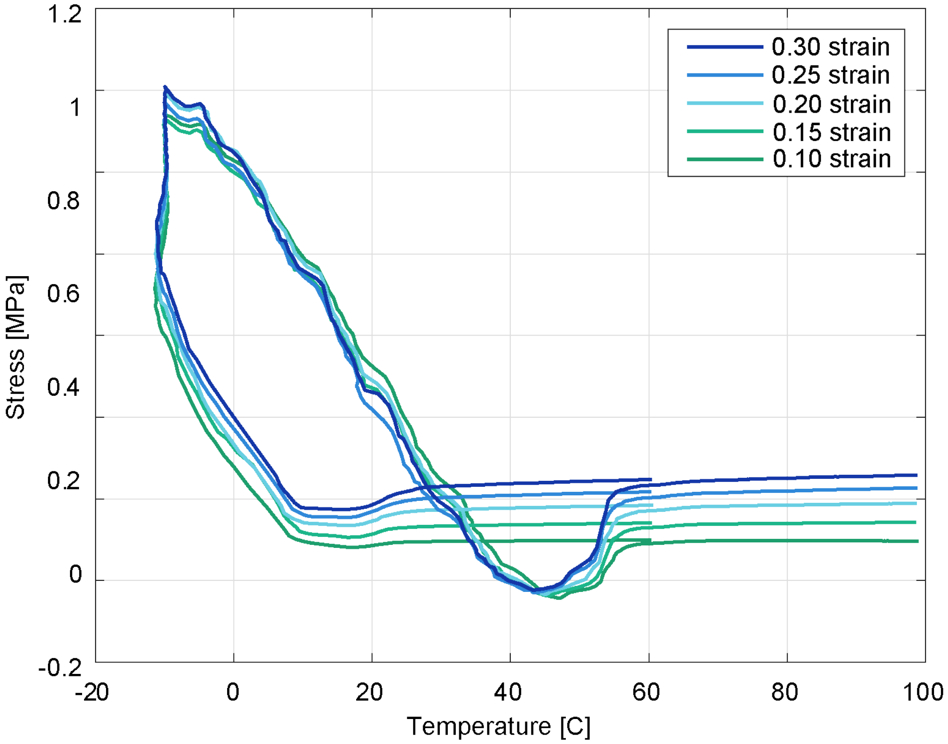

Figure S14: Stress vs. temperature plots at the indicated constant strain value for PCOE membrane sample prepared as in *Fig. S1*. Thermal cycling begins at 60 °C, followed by cooling to -15 °C, and heating to 100 °C.

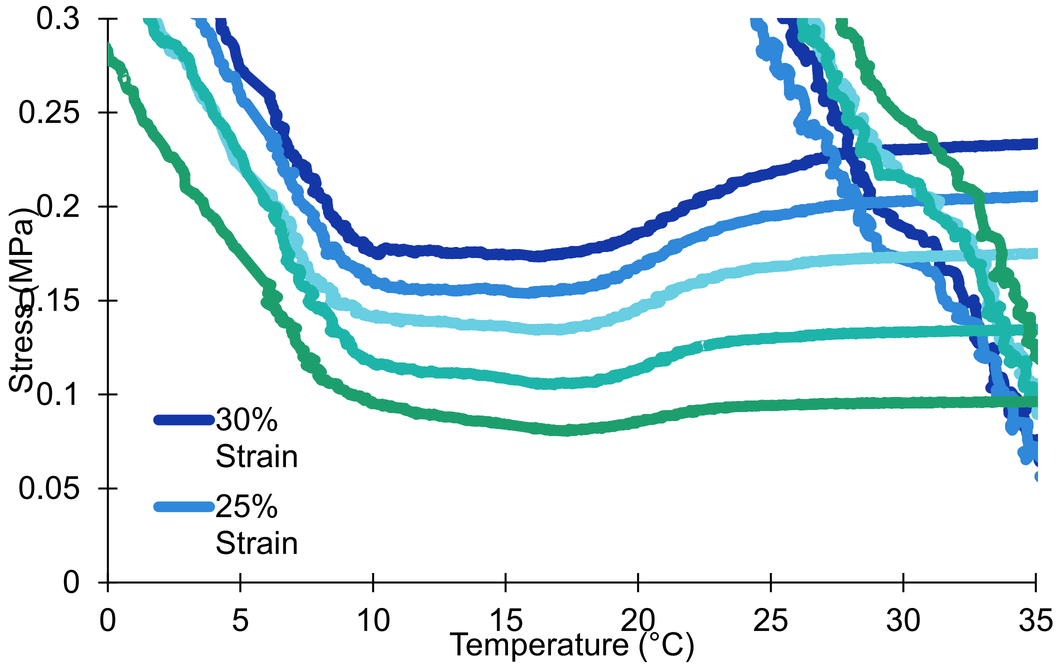

Figure S15: Stress vs. temperature plots at the indicated constant strain value for PCOE membrane sample prepared as in *Fig. S1*. Zoomed in from figure above to highlight the drop in stress upon crystallization.

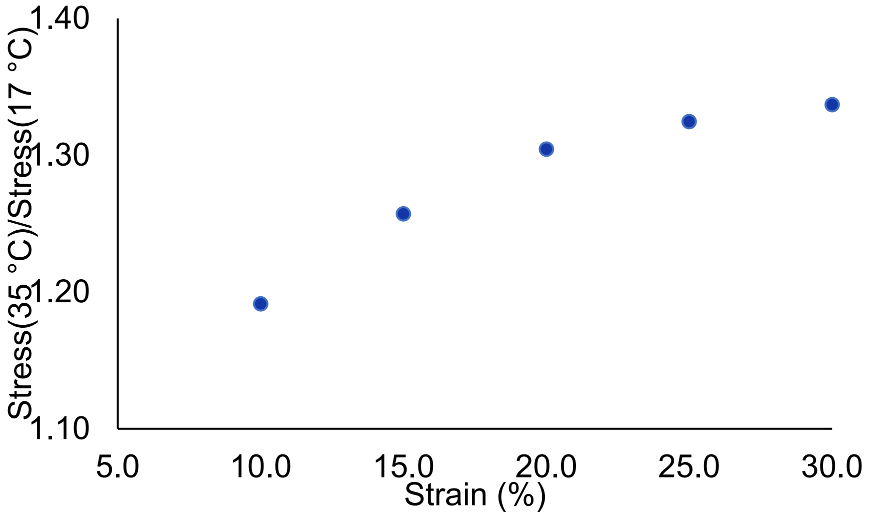

Figure S16: Ratio of stress at 35 °C to stress at 17 °C as a function of % strain. Increasing ratio indicates that the minimum stress in the first stage of crystallization is lower relative to the stress in the melt state, consistent with a larger two-way shape memory effect at higher constant strain values.

The two stage crystallization model proposed by Posada-Murcia and colleagues[3] for cross-linked polycaprolactone involves a first stage where crystallites orient along the axis of the applied stress, resulting in a stress drop at constant strain as the T_c is approached, as we observe here in Figure S15. The

magnitude of the constant strain imposed on the sample affects the magnitude of this stress drop, as quantified by taking the ratio of stresses at 35 °C and 17 °C as in Figure S16. The increasing ratio as a function of strain indicates a larger 2w-SME. In addition, the onset of the stress drop occurs at higher temperatures with higher % strain. A similar trend in the magnitude and position of the stress drop was observed previously in cross-linked polycaprolactone by Posada-Murcia and colleagues.[4]

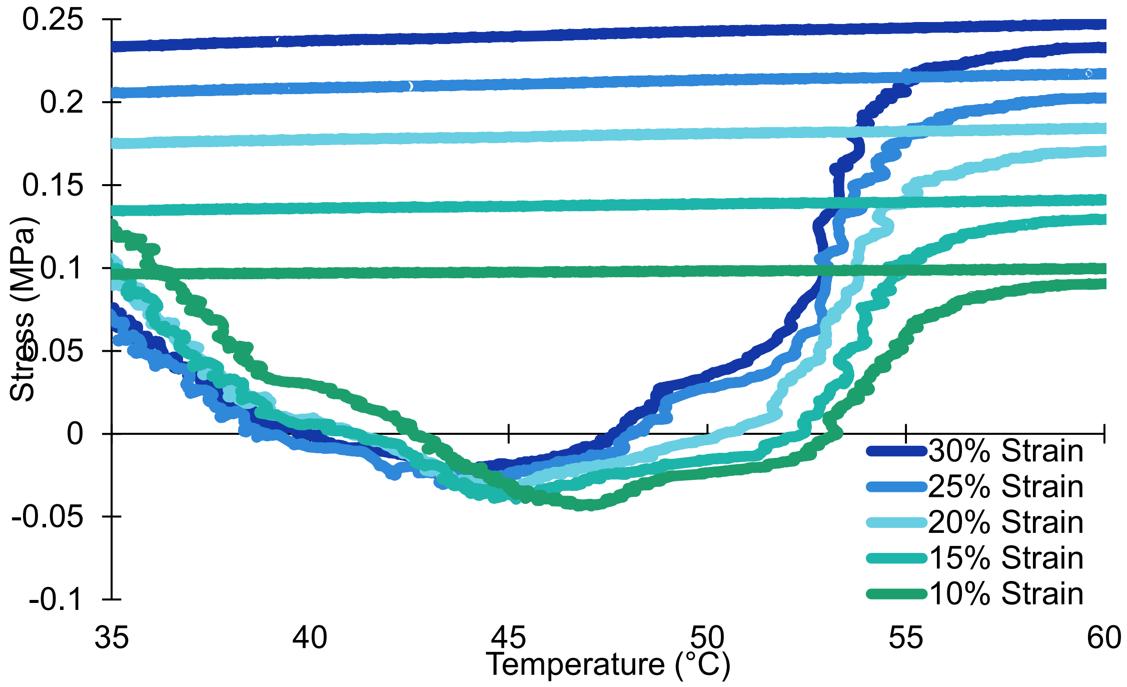

***Figure S17**:* Stress vs. temperature plots at the indicated constant strain value for PCOE membrane sample prepared as in *Fig. S1*. Zoomed in from figure above to highlight the shift in minimum stress upon melting.

The isothermal strain ramp segment at 60 °C was analyzed to plot stress vs. strain from each independent measurement on the same sample.

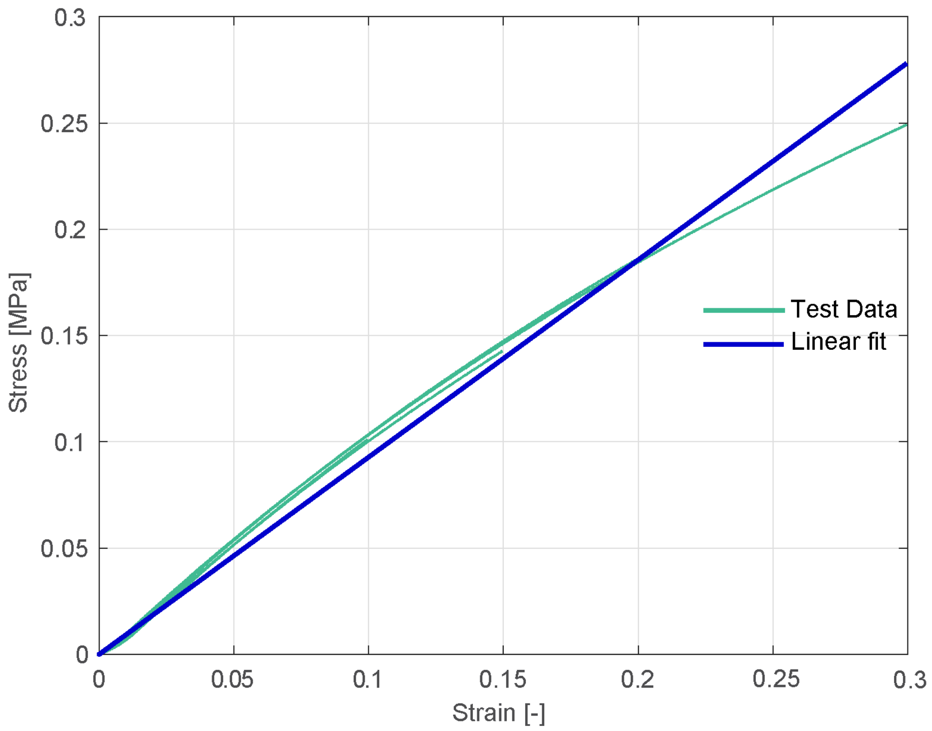

Figure S18: Stress vs. strain (10, 15, 20, 25, and 30% runs) with for PCOE membrane sample prepared as in *Fig. S1,* with linear fit corresponding to $E = 0.9\ MPa,\ R^2 = 0.98$.

7. UV-visible Spectroscopy

A Cary 60 spectrometer (Agilent) was used for UV-visible spectroscopy in double beam mode with an integration time of 0.1 s and a scan rate of 600 nm/min across a range from 300 nm to 800 nm. For kinetic analysis, 400 nm was used as the wavelength which was monitored over time since signals there were stronger than at longer wavelengths. Samples were mounted on glass slides which were affixed to the aperture through which incoming white light enters the sample chamber. A baseline correction was applied to all data, where the baseline was acquired using a glass slide without a sample attached.

For kinetic experiments, the sample was heated to 60 °C as measured with a data logging multimeter (Southwire 15190T) equipped with a type K thermocouple taped to the sample surface with polyimide tape (Kapton). Heating was performed using a heat gun (Porter Cable) adjusted to a low heat setting. Upon reaching 60 °C, the sample was quickly mounted in the instrument and the kinetic run was started. Immediately, the multimeter data collection was started. The sample was allowed to equilibrate to room temperature naturally, while monitoring temperature and UV-vis signal (optical density).

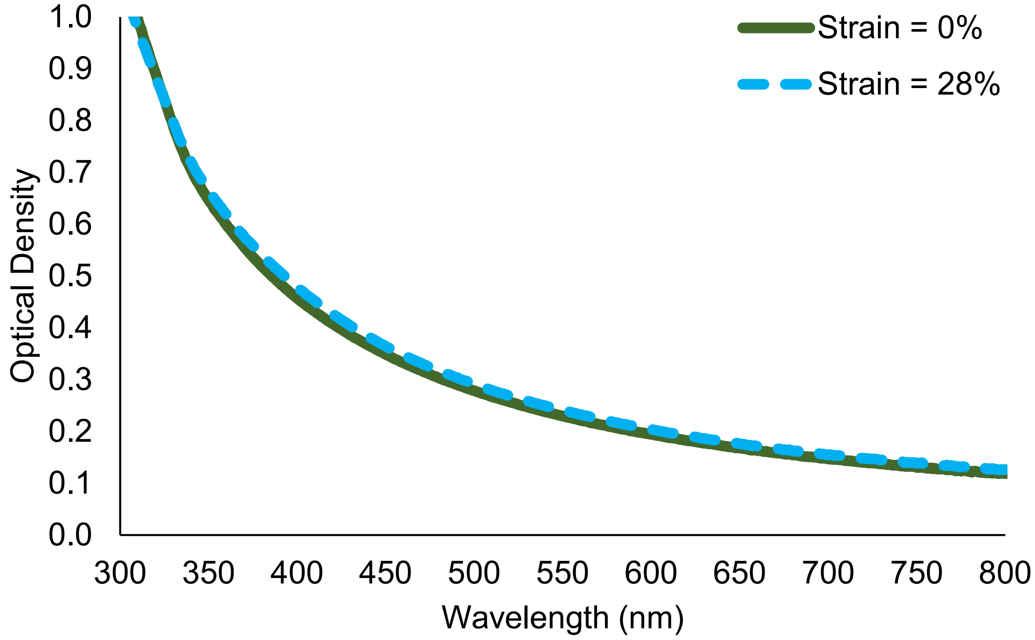

Figure S19: UV-vis spectral trace of PCOE membrane sample prepared as in *Fig. S1,* either pre-strained to 28% or unstrained.

These spectra show a characteristic rise as the wavelength decreases toward the ultraviolet region which is consistent with Rayleigh type light scattering. There is a very small and likely insignificant difference between strained and unstrained samples of 0.021 OD at λ = 400 nm, the wavelength used for the following kinetic study.

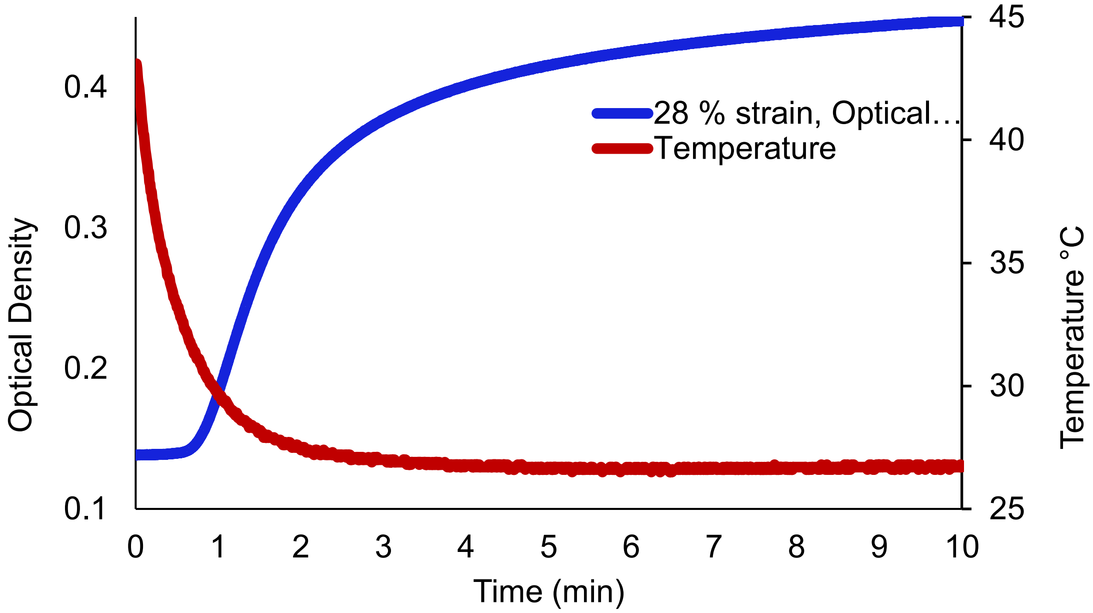

Figure S20: UV-vis kinetic trace of PCOE membrane sample prepared as in Fig. S1, pre-strained uniaxially to 28%, monitored at λ = 400 nm. Temperature was concurrently monitored via a thermocouple mounted on the glass slide.

The optical density is relatively constant, increasing with a small magnitude for ~45 seconds due to the membrane being in the melt state. This small increase in OD may be correlated with the formation of crystallites aligned with the strain axis. Further studies would be needed to confirm this hypothesis.

Once the sample approaches T_c, the OD begins to rise rapidly due to light scattering that can be assigned to bulk crystallization. The onset of rapid increase in OD occurs at ~32-33 °C which corresponds well to the onset of crystallization as measured via DSC.

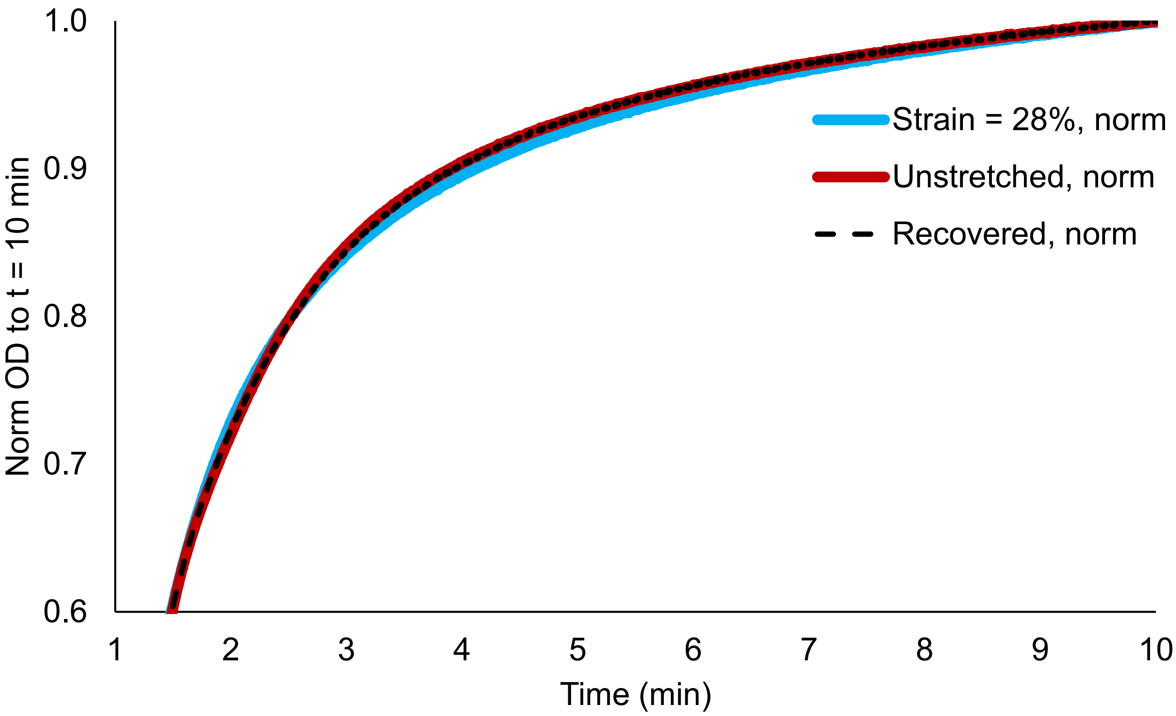

Figure S21: UV-vis kinetic trace of PCOE membrane sample prepared as in Fig. S1, monitored at λ = 400 nm either pre-strained to 28%, unstrained, or recovered.

The 28% strain sample was removed from the glass slide, heated to 60 C, and allowed to undergo shape recovery. Upon cooling to room temperature, the sample was re-mounted and monitored by UV-vis according to the procedure above ("recovered" trace in above figure). In this zoomed-in segment, it can be seen that the recovered sample crystallization kinetics are identical to the unstrained sample. In contrast, a difference in signal was observed with the strained sample in which a faster earlier rise was detected, followed by a slower rise. These changes in signal correspond to changes in light scattering as crystallites form within the polymer upon cooling. The observed difference in kinetics is consistent with the two-step crystallization behavior observed by Posada-Murcia in polycaprolactone crosslinked by DCP,[3] where the early rise observed here may be due to fast crystallization of polymer chains aligned with the strain axis, followed by a slower rise due to reduced population size of the randomly oriented crystallites. Further kinetic studies would be required to test this hypothesis.

8. Uniaxial Tensile Testing

Tensile testing was conducted using a UStretch instrument (CellScale) equipped with a 2.5 N load cell. Each time the instrument was started, the absolute 0 position of the actuator was calibrated by positioning the tines overlapping with the aid of the camera (DMK, Computar macro 10x lens). The position where points of the tines overlap was set as the 0 point. Prior to mounting each sample, the load cell was balanced. To avoid overloading the load cell, a distance experiment was performed with the first sample at each temperature to determine the displacement at which 2.000 N of force is measured. The sample was pulled with a duration of 10 s until the load was reached, at which point the load was held for 120 seconds. This distance was utilized under displacement control in subsequent experiments to obtain the force/displacement curves. A fresh sample was used for each run, and a pre-load of 0.03 N was utilized on the first cycle of each run to ensure the sample was under tension at position = 0. Each run consisted of the following program.

1. Preload to 0.03 N.
2. Ramp to displacement = X in 60 seconds.
3. Ramp to displacement = 0 in 60 seconds.
4. Rest for 10 seconds.
5. Repeat 2-4 for two additional cycles.

Data was acquired at 5 Hz and images recorded at 0.1 Hz. The data for cycles 2 and 3 was averaged and used to calculate Young's Modulus. Samples were approximately 42 mm x 5 mm x 0.18 mm rectangular membranes. For sample synthesis, see section 6. See below for a photograph of typical setup with a sample mounted in the water bath prior to running. The black thermocouple was used to set the bath temperature and the blue (connected to Southwire multimeter) was used to monitor the temperature closer to the sample.

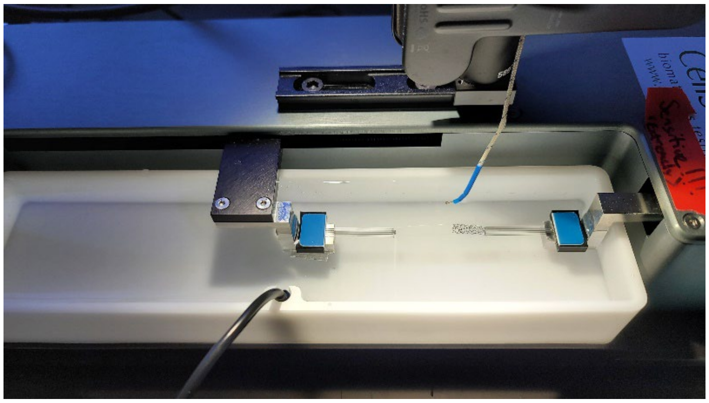

Figure S22: *Photograph of a sample ready for testing in the tensile setup.*

Four samples were run at the temperatures of [10, 22, 42]° C. The second cycle for those samples is reported in the Figures S23-S25, with the respective linear fit and the corresponding young modulus. Table S3 reports a summary of the results.

Table S4: Young's Modulus of the cross-linked PCOE membrane. R^2 is the coefficient of determination. Linear fit parameters from tensile tests illustrated in Figures S18, S23-S25.

Temperature **(°C)**	**E [MPa]**	$\mathbf{R^2}$
10	80.9	0.99
22	62.5	0.99
42	15.9	0.98
60	0.9	0.98

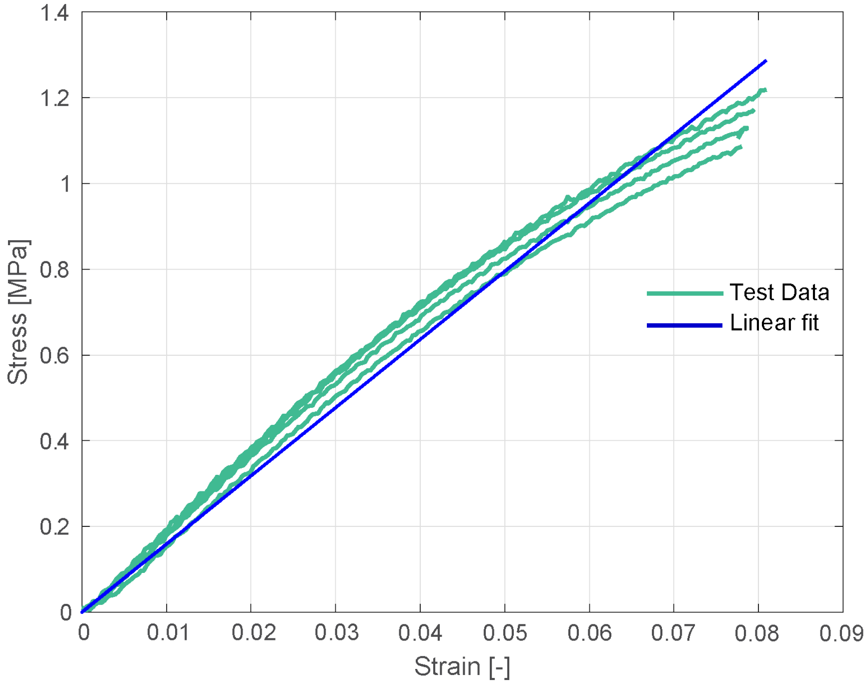

Figure S23: Stress vs. strain curve with for PCOE membrane sample prepared as in *Fig. S1 at 42° C. Linear fit corresponding to* $E = 15.9\ MPa,\ \ R^2 = 0.98$.

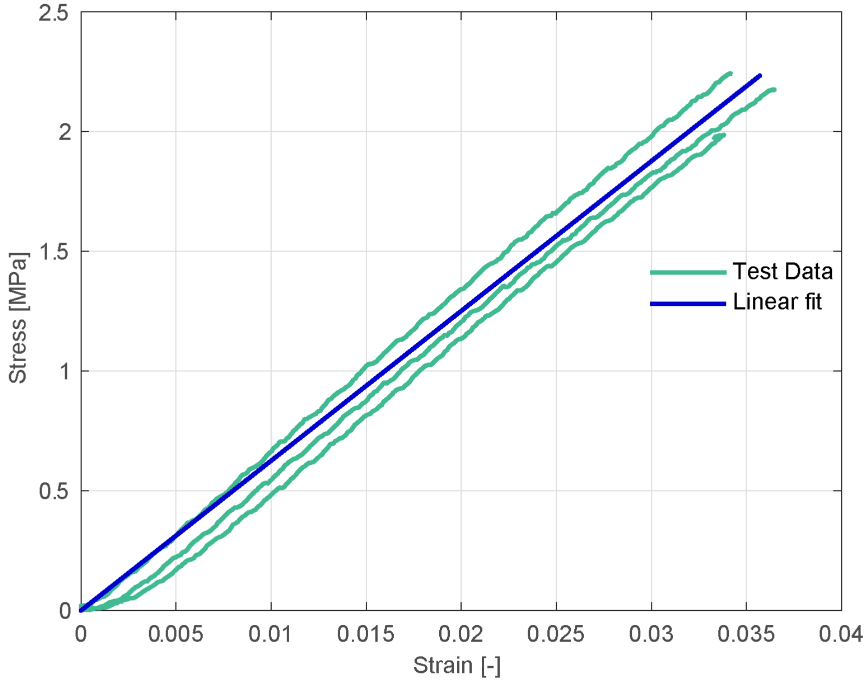

Figure S24: Stress vs. strain curve with for PCOE membrane sample prepared as in *Fig. S1 at 22° C. Linear fit corresponding to* $E = 62.5\ MPa,\ \ R^2 = 0.99$.

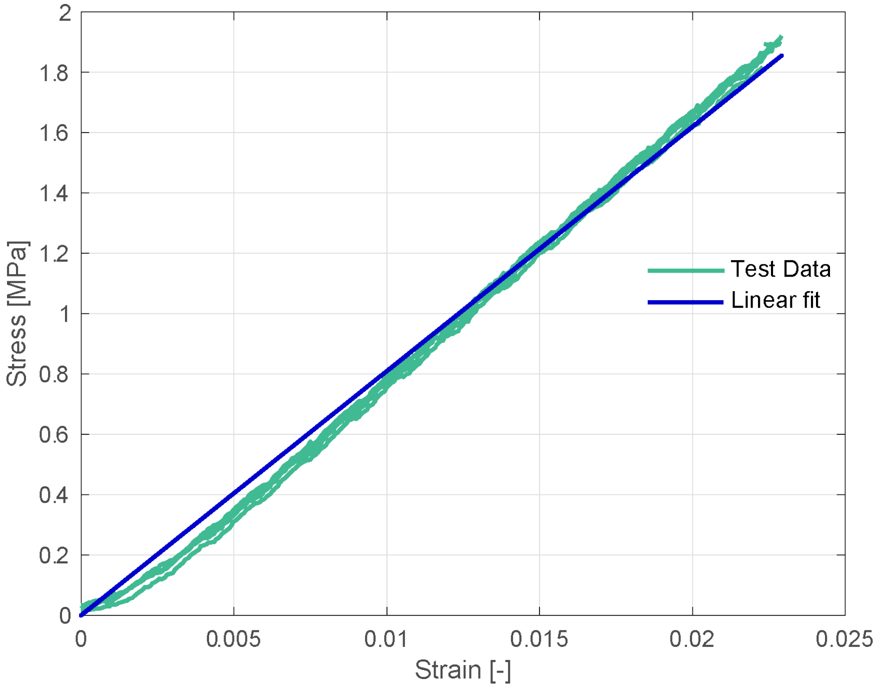

Figure S25: Stress vs. strain curve with for PCOE membrane sample prepared as in *Fig. S1 at 10° C. Linear fit corresponding to* $E = 80.9\ MPa,\ \ R^2 = 0.99$.

9. Membrane Raw Material Synthesis

For all membranes used to construct multi-stable structures, the following procedure was utilized.

To a 1 L round bottom flask charged with a PTFE stir bar was added poly(*cis*-cyclooctene) (Vestenamer 8012, Evonik, 50.000 g). Next, dibenzoyl peroxide (DBzP) was added (2.5 wt. % relative to poly(cis-cyclooctene, multiplied by 1.333 to account for the weight of water present as a stabilizer, 0.360 g), followed by addition of 500 mL toluene under air at room temperature. The mixture was stirred for 48 hours at which point it was noted to be a cloudy viscous solution. The solvent was removed by rotary evaporation at 60 °C during which the solution was noted to become clear and homogeneous. Following removal of the bulk of the solvent, which was collected and reused for additional repeats of this procedure, the solid sample was dried for 48 hours on a Schlenk line (vacuum < 200 mTorr) to remove residual toluene. The dried polymer puck was cut into small chunks with a flush cutter, and the chunks were further dried on the Schlenk line for 24 hours. The dried chunks were processed further as described in Section 1 to create membrane samples except for the cases described below.

A smaller scale synthesis was utilized to create material with dibenzoyl peroxide (DBzP) as crosslinker for DSC kinetic studies varying crosslinking time.

To a 40 mL scintillation vial charged with a PTFE stir bar was added poly(*cis*-cyclooctene) (Vestenamer 8012, Evonik, 3.000 g). Next, dicumyl peroxide was added (2.5 wt. % relative to poly(cis-cyclooctene, multiplied by 1.333 to account for the weight of water present as a stabilizer, 0.100 g), followed by addition of 30 mL toluene under air at room temperature. The mixture was stirred for 48 hours at which point it was noted to be a clear homogeneous solution. The solvent was removed by rotary evaporation at 60 °C. Following removal of the bulk of the solvent, which was collected and reused for additional repeats of this procedure, the solid sample was dried for 48 hours on a Schlenk line (vacuum < 200 mTorr) to remove residual toluene. The dried polymer puck was cut into small chunks with a flush cutter, and the chunks were further dried on the Schlenk line for 24 hours. The dried chunks were processed further as described in the DSC Analysis Section above.

A smaller scale synthesis was utilized to create material with dibenzoyl peroxide (DBzP) as crosslinker for DSC studies varying crosslinker loading.

To a 20 mL scintillation vial charged with a PTFE stir bar was added poly(*cis*-cyclooctene) (Vestenamer 8012, Evonik, 1.000 g). Next, dicumyl peroxide was added (1.0-4.0 wt. % relative to poly(cis-cyclooctene, multiplied by 1.333 to account for the weight of water present as a stabilizer, 0.013-0.053 g), followed by addition of 1.0 wt. % (0.010 g) hollow glass microspheres and 10 mL toluene under air at room temperature. The mixture was stirred for 48 hours at which point it was noted to be a clear homogeneous solution. The solvent was removed by rotary evaporation at 60 °C. Following removal of the bulk of the solvent, which was collected and reused for additional repeats of this procedure, the solid sample was dried for 48 hours on a Schlenk line (vacuum < 200 mTorr) to remove residual toluene. The dried polymer puck was cut into small chunks with a flush cutter, and the chunks were further dried on the Schlenk line

for 24 hours. The dried chunks were processed further by placing them in a cylindrical PTFE mold heated to 70 °C in an oven to melt the raw polymer without triggering the crosslinking reaction. Polymer was added until there was a small mound of excess material spilling out of the mold. The mold was compressed while hot with a C clamp between two PTFE plates to remove bubbles and excess material. The clamped assembly was then placed in a vacuum oven (VWR Symphony) and heated to 90 °C for 43 hours to complete the crosslinking reaction. Upon returning to room temperature, the resulting cylinders (~5.0 mm diameter, ~6.3 mm height) were removed from the mold and a small piece was cut away for DSC analysis.

A smaller scale synthesis was utilized to create material with dicumyl peroxide (DCP) as crosslinker for DSC studies varying DCP loading.

To a 20 mL scintillation vial charged with a PTFE stir bar was added poly(*cis*-cyclooctene) (Vestenamer 8012, Evonik, 1.000 g). Next, dicumyl peroxide was added (1.0-2.0 wt. % relative to poly(cis-cyclooctene, 0.01-0.02 g), followed by addition of 10 mL toluene under air at room temperature. The mixture was stirred for 48 hours at which point it was noted to be a clear homogeneous solution. The solvent was removed by rotary evaporation at 60 °C. Following removal of the bulk of the solvent, which was collected and reused for additional repeats of this procedure, the solid sample was dried for 48 hours on a Schlenk line (vacuum < 200 mTorr) to remove residual toluene. The dried polymer puck was cut into small chunks with a flush cutter, and the chunks were further dried on the Schlenk line for 24 hours. The dried chunks were processed further by placing them in a cylindrical PTFE mold heated to 70 °C in an oven to melt the raw polymer without triggering the crosslinking reaction. Polymer was added until there was a small mound of excess material spilling out of the mold. The mold was compressed while hot with a C clamp between two PTFE plates to remove bubbles and excess material. The clamped assembly was then placed in a vacuum oven (VWR Symphony) and heated to 150 °C for 43 hours to complete the crosslinking reaction. Upon returning to room temperature, the resulting cylinders (~5.0 mm diameter, ~6.3 mm height) were removed from the mold and a small piece was cut away for DSC analysis.

10. Actuation Work Testing

To measure the specific work of actuation, a modified procedure was followed based on a literature method.[5] A thin strip was cut from a membrane prepared as described in Section 1: containing 2.5% DBzP and crosslinked in the vacuum hot press setup. The strip was heated to 70 °C in an oven and stretched by hand while hot to its maximum strain without breakage (approx. 200%). The strip was measured to have a length of 39.23 mm with electronic calipers and weight of 17.1 mg. A fixed clamp was attached to one end of the strip and a clamp (weight 104.2 g) was attached to the free end (see photograph below). The strip was noted to hold the clamp without significant change in strain. Using a heat gun, the strip was heated to 70 °C during which it was noted to contract to a minimum recovered length of 27.28 mm. Upon turning off the heat gun and allowing the strip to crystallize with the weight attached, significant elongation was noted, with the strip returning to stretched length of 35.90 mm. Repeated actuation

cycling became possible due to the two-way shape memory effect, where the strip would contract upon heating the melt state and elongate during crystallization.

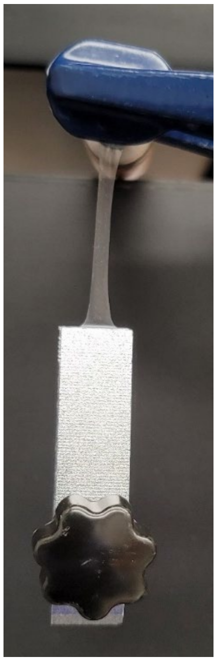

Figure S26: A photograph of the setup for the actuation test showing the membrane strip with clamped fixed end (top) and free end holding a clamp as a weight.

The specific work of actuation W was calculated to be 0.71 J g^{-1} utilizing the height the clamp was lifted during actuation ($h_a = 11.95$ mm), the ratio of lifted weight to strip weight ($\frac{m_i}{m_a} = 104200$), and the acceleration due to gravity ($g = 9.81$ m s^{-2}) according to the following equation: $W = \frac{m_i}{m_a} g h_a$. We note that this specific work compares favorably (within a factor of 3) with that of the material developed by Bao & coworkers that is among the highest energy density of reported actuators.[5]

11. Composite Material Details

A unit cell consisted of four identical rectangular strips manufactured employing HR40/513 epoxy prepreg from NTPT and a [0/90/0] layup. Details about the material are given in Table S4.

Table S5: Y Material properties for NTPT Thinpreg 513/HR40 carbon fiber-reinforced prepreg used to manufacture the experimental specimens [3, 4].

E_{11} [MPa]	E_{22} [MPa]	G_{12} [MPa]	ν_{12} [-]	t_{ply} [mm]	v_f [-]
232000	7010	4661	0.31	0.045	0.6

12. Tailoring the out-of-plane deformation of FRP frames

To decrease the deformations of a frame in its stable state with four inflection points, the maximum height of the frame can be constrained by placing the structure during the cooling phase between two rigid plates kept at a fixed distance from one another. Figure S27 illustrates the experimental setup with a structure between the two rigid plates and the spacers. Figure S28 shows three examples of the frame with 25% pre-stretching with different out-of-plane deformations obtained with this method. The frames are fixed at an out-of-plane deformation of 15, 10, and 7 mm respectively and they possess multi-stability. No change in out-of-plane deformation was observed after removing the structures from the plates. Structures with an out-of-plane deformation lower than 7 mm did not show multi-stability.

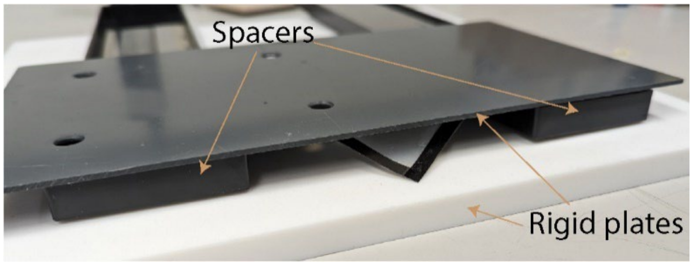

Figure S27: Experimental setup to tailor the out-of-plane deformation of FRP cells.

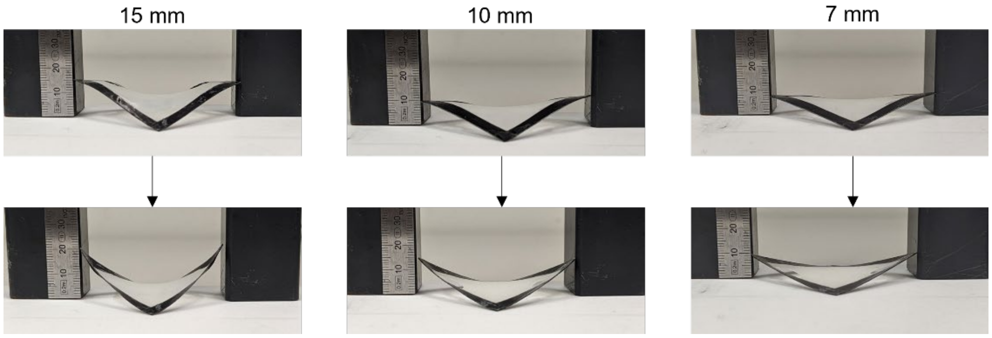

Figure S28: The out-of-plane deformation of the structure can be increased/decreased by imposing external constraints during crystallization.

13. Description of the multi-stability of periodic structures

In this section, we provide a summary of the multi-stability property of periodic structures at room temperature. The reader is invited to refer to previous literature [6] for further details. The stable state in Figure S29a is characterized by a total of four inflection points per unit cell. This state is the one that appears once the boundary-free shape forming is realized. Another possible configuration is illustrated in Figure S29b: here, every unit cell has zero inflection points and in this state the surface forms a cylinder with the axis along the diagonal of the grid. The structure is also stable when each unit cell has a total of two inflection points. Furthermore, many more stable configurations can be obtained by combining unit cells with either four, two, or no inflection points, an example is shown in Figure S29d. We define these types of stable states as "mixed mode". Importantly, we observe that to have cells with strips with zero inflection points, the deformation of each unit cell must be identical along diagonal lines of the grid. Figure

S29 shows a schematic illustrating with red lines the diagonal lines along which the strips have zero inflection points. This schematic is identical to the one utilized in previous work [6].

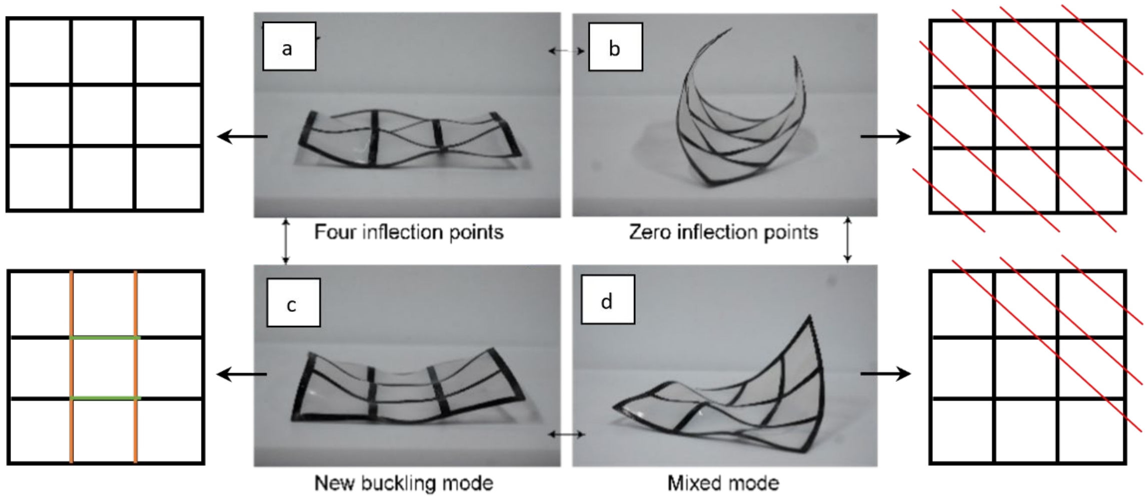

Figure S29: *Four stable states of a 3x3 structure at room temperature.*

Moreover, a unit cell in periodic architectures has a stable state that is not possible to obtain with a single frame. This new configuration is illustrated in Figure S29c. In this case, a single FRP strip of the unit cell in the center of the structure buckles with two inflection points, with a deformation like one of buckled beams under compression with both edges clamped. Figure S30 shows in detail the deformation of a unit cell [6]. Not all four strips have the same buckled shape, only two opposite strips possess two inflection points, while the other two have none. The configuration in Figure S29c forms a cylinder with principal axes along the grid lines. This state is realized by applying an out-of-plane force to one or multiple intersection points of the grid, following horizontal or vertical lines (highlighted for example in orange in the schematic of Figure S29c). Given the possible combination of stable states that can co-exist, a central cell (i.e., not at the edges of the structure) in a periodic possesses 9 different stable states [6]. The cells that are located at the edges of a periodic structure possess a lower number of achievable shapes because they have different boundary conditions. One can observe that the FRP strips of the cells in the internal area of the structure intersect one another with an X-intersection, while the ones at the edges have L- or T-like intersections.

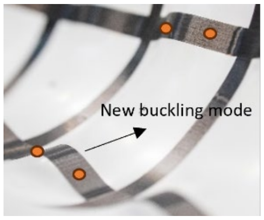

Figure S30: *example of a unit cell in the new buckling mode of stability*

Besides the stable state thus far described, the hybrid structures can be shaped into new configurations via programming. This implies that one can “freeze” the structure into a new 3D shape and temporarily suppress its multi-stability. If a single cell can be shaped into a saddle or be completely flat, in periodic structures, the user can shape either one, multiple, or all cells into a flat or saddle shape, or combinations of those. An example is shown in Figure S31, where Figure S31a shows a structure with all cells in a saddle configuration, Figure S31b all cells flat, and Figure S31c a combination of those. Importantly both the FRP strips and membrane must be maintained in their linear regime to guarantee reversibility and re-usability.

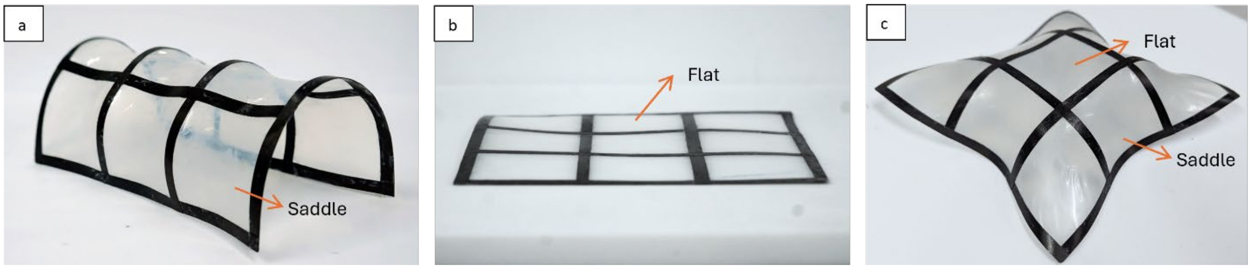

Figure S31: *Three examples of temporary shapes that can be imposed via programming.*

14. Bibliography

1. Kudisch, M.; Ko, J. K.; Daraio, C.; Shapiro, M. G.; Wu, D.; Kim, G.; Grubbs, R. H.; Yoon, K.-Y. Ultrasound Responsive Shape Memory Polymer Composites. US20230219282A1, **2023**.

2. Chung, T.; Romo-Uribe, A.; Mather, P. T., Two-Way Reversible Shape Memory in a Semicrystalline Network. *Macromolecules* **2008**, 41 (1), 184-192.

3. Posada-Murcia, A. P.; Uribe-Gomez, J. M. U.; Förster, S.; Sommer, J.-U.; Dulle, M.; Ionov, L., Mechanism of Behavior of Two-Way Shape Memory Polymer under Constant Strain Conditions. *Macromolecules* **2022**, 55 (5), 1680-1689.

4. Posada-Murcia, A. P.; Uribe-Gomez, J. M. U.; Sommer, J.-U.; Ionov, L., Two-Way Shape Memory Polymers: Evolution of Stress vs Evolution of Elongation. *Macromolecules* **2021**, 54 (12), 5838-5847.

5. Cooper, C. B.; Nikzad, S.; Yan, H.; Ochiai, Y.; Lai, J.-C.; Yu, Z.; Chen, G.; Kang, J.; Bao, Z., High Energy Density Shape Memory Polymers Using Strain-Induced Supramolecular Nanostructures. *ACS Central Science* **2021**, 7 (10), 1657-1667.

6. G. Risso, M. Sakovsky, P. Ermanni, A highly multi-stable meta-structure via anisotropy for large and reversible shape transformation, Advanced Science **2022**, 9 (26) 2202740
